# Perceptual Discrepancies of Opioid Analgesics and Psychotropic Drugs: A Cross-Sectional Study of Korean Patients and Physicians

**DOI:** 10.3390/jcm14217734

**Published:** 2025-10-31

**Authors:** Yongsoo Lee, Eun Hee Chun, Yang-Ki Minn, So Hyun Ahn, Jae Hun Kim, Hee Yong Kang, Hye Sun Lee, Jung Eun Kim

**Affiliations:** 1 Department of Anesthesiology and Pain Medicine, Hallym University Kangnam Sacred Heart Hospital, Seoul 07441, Republic of Korea; thisisrio@naver.com (Y.L.);; 2 Department of Neurology, Hallym University Kangnam Sacred Heart Hospital, Seoul 07441, Republic of Korea; 3 Department of Anesthesiology and Pain Medicine, Konkuk University School of Medicine, Seoul 05030, Republic of Korea; 4 Department of Anesthesiology and Pain Medicine, Kyung Hee University College of Medicine, Seoul 02447, Republic of Korea; 5 Biostatistics Collaboration Unit, Yonsei University College of Medicine, Seoul 03722, Republic of Korea

**Keywords:** narcotics, illicit drugs, opioid analgesics, perception, psychotropic drugs, narcotics information management system, prescription drug monitoring programs, drug and narcotic control

## Abstract

**Background:** Opioid analgesics and psychotropic drugs (medical narcotics) are essential for treating pain and psychiatric disorders. Unlike tiered classification systems used globally, Korea uniformly classifies these medications with illicit drugs under a single narcotics category. This creates misunderstandings among patients and physicians. This study investigates perceptions of medical narcotics, assesses awareness of the Narcotics Information Management System (NIMS), and proposes strategies to prevent misuse and abuse. **Methods**: A cross-sectional survey from September 2021 to June 2025 enrolled 322 patients prescribed opioid analgesics or psychotropic drugs for ≥180 days per year and surveyed 300 physicians via email. Categorical variables were expressed as frequencies (percentages) and compared using a Chi-square test. Multivariable logistic regression adjusted for age and gender was performed and subgroup analyses were performed, including by patient education level (using ANOVA and Bonferroni-corrected post hoc comparisons) and treatment duration, alongside physician specialty and affiliation. **Results**: Significant perception differences emerged between patients and physicians. The largest perception discrepancy was in distinguishing medical narcotics from illicit drugs (48.8 percentage point difference; 9.9% vs. 58.7%; *p* < 0.001). The NIMS Data Service awareness was lowest in both groups (patients 14.6% vs. physicians 34.3%). In multivariable-adjusted analysis, perception differences were greater in those over 60 years old. In the subgroup analysis, patient-physician perception gaps, as reflected by odds ratios, were greater in patient-groups with shorter treatment duration (<36 months) compared to those with longer treatment duration (≥36 months). **Conclusions**: Perceptions of opioid analgesics and psychotropic drugs are significantly different between patients and physicians. Both groups showed limited awareness of medical narcotics and the narcotics control system. Targeted educational initiatives are crucial for both patients and physicians to bridge existing perceptual and knowledge gaps, especially for patients aged 60 years or older and patients with shorter medical narcotics treatment duration (less than 36 months).

## 1. Introduction

Opioid analgesics and psychotropic drugs are essential pharmacotherapies for managing both acute and chronic pain, as well as various psychiatric disorders [1,2,3]. These agents exert therapeutic effects through central nervous system modulation, providing analgesia, anxiolysis, and sedation [4]. In South Korea, the Narcotics Control Act uniformly regulates medical narcotics (including opioid analgesics and psychotropic drugs), cannabinoids, and illicit drugs (Korean: *mayak*; literally ‘drugs’) under a single legal category termed narcotics (Korean: *mayak-ryu*) [5]. Pharmacologically, these centrally acting agents can induce analgesia, anesthesia, altered states of consciousness, hallucinations, and physical or psychological dependence [6]. However, medical narcotics such as opioid analgesics and psychotropic drugs are clinically necessary and should be clearly distinguished from illicit drugs [2,7]. This uniform regulatory framework creates substantial clinical barriers by causing patients to conflate prescribed medical narcotics with illicit drugs, leading to treatment non-adherence due to fear of social stigma and therapeutic misconceptions [8,9]. Supporting this concern, in a multicenter survey of pain clinics in South Korea, 39.0% of patients reported refusing to take prescribed opioid analgesics, primarily due to fear of addiction (57.7%) and side effects (38.5%) [9].

While most countries employ tiered classification systems that differentiate medical narcotics by abuse potential, the Narcotics Control Act in South Korea regulates both medical narcotics and illicit drugs as a single class of narcotics [5,10,11]. This uniform classification, combined with the linguistic similarity between the terms for narcotics (*mayak-ryu*) and illicit drugs (*mayak*), leads to widespread misunderstanding among patients and physicians [8,12]. Furthermore, this linguistic confusion, compounded by media coverage of illicit drug abuse by celebrities, has intensified public concern and negative perceptions toward medical narcotics [13,14]. Consequently, these factors undermine treatment adherence and weaken the therapeutic relationship between patients and physicians [8,9].

To ensure the safe prescription of medical narcotics, the South Korean government has implemented a comprehensive narcotics control system, the Narcotics Information Management System (NIMS) and NIMS Data Service, which is modeled after international monitoring systems, particularly the United States (U.S.) Prescription Drug Monitoring Program (PDMP) [15]. Despite these monitoring systems, both patients and physicians demonstrate limited awareness of the system and its functions, thereby complicating the optimal treatment process [15,16].

While previous studies have primarily focused on patient awareness of prescription medication misuse, to our knowledge, no prior studies have concurrently assessed the perceptions of patients and physicians in this regulatory context [17,18,19]. Particularly in South Korea’s unique regulatory framework, where medical narcotics and illicit drugs are uniformly classified under a single act, direct comparison of patient and physician viewpoints is essential to identify the causes of clinical barriers.

Therefore, this study aimed to (1) assess patients’ and physicians’ perceptions of opioid analgesics and psychotropic drugs; (2) evaluate awareness of the narcotics control system including the NIMS and NIMS Data Service in both groups, with stratified analyses by age and other key characteristics; (3) identify and quantify perceptual gaps regarding narcotics misuse and abuse; and (4) propose evidence-based strategies to optimize safe and effective prescription of medical narcotics.

## 2. Materials and Methods

### 2.1. Study Design and Ethical Approval

This cross-sectional study was approved by the Institutional Review Board of Hallym University Kangnam Sacred Heart Hospital (IRB protocol number 2020-09-001; 14 October 2020), before the first participant was enrolled in September 2021. The study was conducted in accordance with the Declaration of Helsinki. Initial data were collected from September 2021 to June 2025. To enhance transparency and public accessibility, the study was registered with the University Hospital Medical Information Network Clinical Trials Registry (UMIN-CTR, UMIN000058401). To further characterize patient demographics and digital health literacy, a protocol amendment was approved by the IRB to conduct follow-up telephone surveys (September–October 2025) with the originally enrolled patients. Supplementary Data on educational attainment and digital health literacy were collected. This cross-sectional study adhered to STROBE guidelines (checklist in Supplementary Materials S3).

### 2.2. Patient Recruitment

We conducted a one-year preliminary review of the NIMS prescription records to identify departments with the highest frequencies of prescriptions for opioid analgesics and psychotropic drugs. Anesthesiology and Pain Medicine, Oncology, Neurology, and Psychiatry emerged as the highest-frequency departments, from which we sampled high-exposure clinics to maximize the internal validity of perception comparisons. Oncology was excluded due to distinct cancer-pain context, anticipated feasibility constraints, and ethical considerations regarding continuity of active treatment.

Based on these findings, we recruited 400 outpatients from the departments of Anesthesiology and Pain Medicine, Neurology, and Psychiatry. Eligibility required prescription of medical narcotics for ≥180 days per year. Patients were excluded if they met any of the following criteria: (1) age under 18 years; (2) use of opioid analgesics or psychotropic drugs for less than 6 months; (3) active prescription of opioid analgesics for cancer-related pain; (4) unstable medical or psychiatric condition documented in the medical record; or (5) declined participation. Of the 400 patients initially recruited, 12.5% (*n* = 50) dropped out, leaving 350 patients included in the final analysis. After obtaining informed consent, 350 patients completed self-administered questionnaires in outpatient clinical settings. The survey primarily consisted of standardized objective-type questions to ensure clarity and consistency in responses (Supplementary Materials S1: Patient Survey).

Between September and October 2025, follow-up telephone surveys were conducted to collect Supplementary Data on educational attainment and digital health literacy (assessed using the Korean version of the eHealth Literacy Scale; K-eHEALS). Of the 350 patients originally enrolled, 322 completed the telephone interviews [20,21]. Trained research assistants conducted all interviews using a standardized script. Verbal informed consent was obtained, and responses were linked to previously collected data via unique identification numbers. The follow-up protocol was approved as an amendment to the original IRB protocol.

### 2.3. Physician Recruitment

Physician email addresses specializing in Anesthesiology and Pain Medicine, Neurology, and Psychiatry were obtained from the Korean Medical Association database. Survey administration was conducted by a professional research company to ensure standardized email delivery and data collection protocols. Individual invitation emails were sent with a one-response-per-physician policy to prevent duplicate submissions. The invitation emails contained detailed study objectives, estimated completion time (5–10 min), data handling procedures, and electronic informed consent forms. Physicians could respond through a secure link provided in the invitation. The survey remained open for 8 weeks, with 800 initial invitations sent; 309 completed the survey. After excluding 9 responses with data quality issues that failed to meet inclusion criteria, 300 physicians were included in the final analysis. All response data were coded with non-identifiable numbers for confidentiality. The questionnaire collected demographic information, including gender, age, medical specialty, department, and affiliation. It also assessed perceptions of medical narcotics, attitudes toward narcotics misuse and abuse, and strategies to mitigate such misuse (Supplementary Materials S2: Physician Survey). Physicians without experience in prescribing medical narcotics were excluded.

### 2.4. Questionnaire Development and Validation

The survey instruments were developed through a multi-step process emphasizing content validity. Following a comprehensive literature review, a six-member specialist panel evaluated content validity using a 4-point relevance scale, yielding a scale-level Content Validity Index (S-CVI/Ave) of 0.87. Cognitive interviews with 20 participants (10 patients, 10 physicians) assessed item clarity and comprehension. Formal pre-study construct validation (e.g., factor analysis) was not performed.

Post hoc internal consistency analysis revealed low Cronbach’s α values for the overall scale (combined α = 0.53) and for conceptual subscales (Knowledge and awareness [Q1–Q3], System accessibility [Q4–Q7], Misuse and abuse [Q8–Q9]; α range: −0.04 to 0.61), indicating items measure distinct facets. Therefore, all items were analyzed independently. Full statistics are provided in Supplementary Table S1. The instruments were developed and administered in Korean; thus, translation was not required. Test–retest reliability was not assessed due to the cross-sectional design.

### 2.5. Sample Size Determination

The sample size was determined using PASS (version 15, NCSS, LLC, Kaysville, UT, USA). Assuming an alpha of 0.05, a power of 0.8, and a 10% difference in the awareness rates between the two groups, the minimum required sample size was approximately 300 participants per group. Considering an anticipated dropout rate of 25% for the patient group, we aimed to recruit 400 patients. For the physician group, with an expected dropout rate exceeding 60%, a target of 800 physicians was set. Ultimately, 322 patients and 300 physicians were included in the final analysis.

### 2.6. Statistical Analysis

Categorical variables were expressed as frequencies (percentages), and compared between patients and physicians using the Chi-square test (Fisher’s exact test). Continuous variables were expressed as mean ± standard deviation, and compared using independent *t*-tests. Multivariable logistic regression analysis was conducted to assess perception gaps between patients and physicians, with adjustment for age and gender. To control for multiple comparisons across the matched survey items administered to both groups, we applied the Benjamini–Hochberg procedure to control the false discovery rate (FDR).

Additional stratified analyses were performed by patient education level (high school or less, associate degree, bachelor’s degree, graduate degree) to assess whether educational attainment influenced survey responses. One-way ANOVA was used to compare response across education groups, followed by Bonferroni-corrected post hoc pairwise comparisons for stratified analysis to control Type I error inflation.

Subgroup analyses were conducted by gender and age (<60 and ≥60), followed by logistic regression within each age stratum. To assess the consistency of the main analysis results, additional subgroup analyses were conducted according to patient treatment duration (6–12 months, >12–36 months, >36–60 months, >60 months), physician specialty (Anesthesiology and Pain Medicine, Neurology, Psychiatry), and physician practice setting (private clinic, employed physician, university hospital). Each patient subgroup was compared with all physician participants, and each physician subgroup was compared with all patient participants. Heterogeneity of effect sizes between subgroups was evaluated by examining overlap of 95% confidence intervals (CI) for odds ratios (OR).

A two-tailed *p*-value of less than 0.05 was considered statistically significant. All statistical analyses were performed using SAS version 9.4 (SAS Institute Inc., Cary, NC, USA) and R version 4.4.1 (R Foundation for Statistical Computing, Vienna, Austria).

## 3. Results

### 3.1. Participant Characteristics

A total of 322 patients (mean age 51.25 ± 15.36 years; 64.0% female) and 300 physicians (49.14 ± 7.34 years; 16.7% female) were included. Patients were significantly older than physicians (*p* = 0.027) and had a significantly higher proportion of females (*p* < 0.001). To account for these baseline differences, age and gender were adjusted in subsequent analyses, including the multivariable logistic regression. Patient and physician demographic and clinical characteristics are presented in Table 1.

### 3.2. Primary Study Outcomes

**Figure 1:**
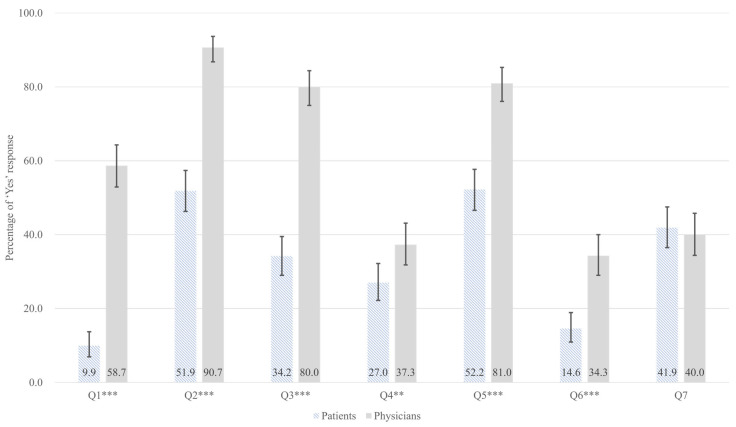
Adjusted predicted probabilities with 95% confidence intervals for ‘Yes’ responses across survey questions (Q1–Q7), comparing patients (hatched bars, *n* = 322) and physicians (gray bars, *n* = 300). Probabilities are adjusted for age and gender using multivariable logistic regression. Error bars indicate 95% confidence intervals. ** *p* < 0.01; *** *p* < 0.001 (FDR-adjusted *p*-values from Chi-square tests). Survey items. Q1: Can you distinguish medical narcotics from illicit drugs?; Q2: Are you aware that your prescribed medications are classified as medical narcotics?; Q3: Are you aware of the Narcotics Information Management System (NIMS) reporting?; Q4: Are you aware of the system for checking a patient’s narcotics prescription history (narcotics prescription status inquiry system)?; Q5: Are you aware of physicians’ right to refuse a prescription?; Q6: Are you aware of the NIMS Data Service?; Q7: Are you willing to try the NIMS Data Service?

The perceptions of patients and physicians regarding opioid analgesics and psychotropic drugs were analyzed, and the results are presented in Table 2 and Figure 1. Of the survey items, seven showed a statistically significant difference between the two groups. In the area of narcotics knowledge and awareness (Q1, Q2, Q3), physicians demonstrated a significantly higher level of awareness compared to patients. The largest gap was observed in the recognition of the distinction between medical narcotics and illicit drugs (Q1, 48.8 percentage points [pp] difference; 9.9% vs. 58.7%; FDR-adjusted *p* < 0.001).

Similarly, physicians showed significantly higher awareness that prescribed medications are classified as medical narcotics (Q2, 38.8 pp; 51.9% vs. 90.7%; FDR-adjusted *p* < 0.001), awareness of the NIMS reporting (Q3, 45.8 pp; 34.2% vs. 80.0%; FDR-adjusted *p* < 0.001). In terms of narcotics control system accessibility, physicians showed significantly higher awareness of the narcotics prescription status inquiry system (Q4, 10.3 pp; 27.0% vs. 37.3%; FDR-adjusted *p* = 0.008) and physician’s right to refuse a prescription (Q5, 28.8 pp; 52.2% vs. 81.0%; FDR-adjusted *p* < 0.001). Additionally, the lowest awareness for both groups was awareness of the NIMS Data Service (Q6), a special service within the NIMS that enables physicians to check patient’s narcotic medication histories, showing a difference of 19.7 pp (14.6% vs. 34.3%; FDR-adjusted *p* < 0.001).

**Figure 2:**
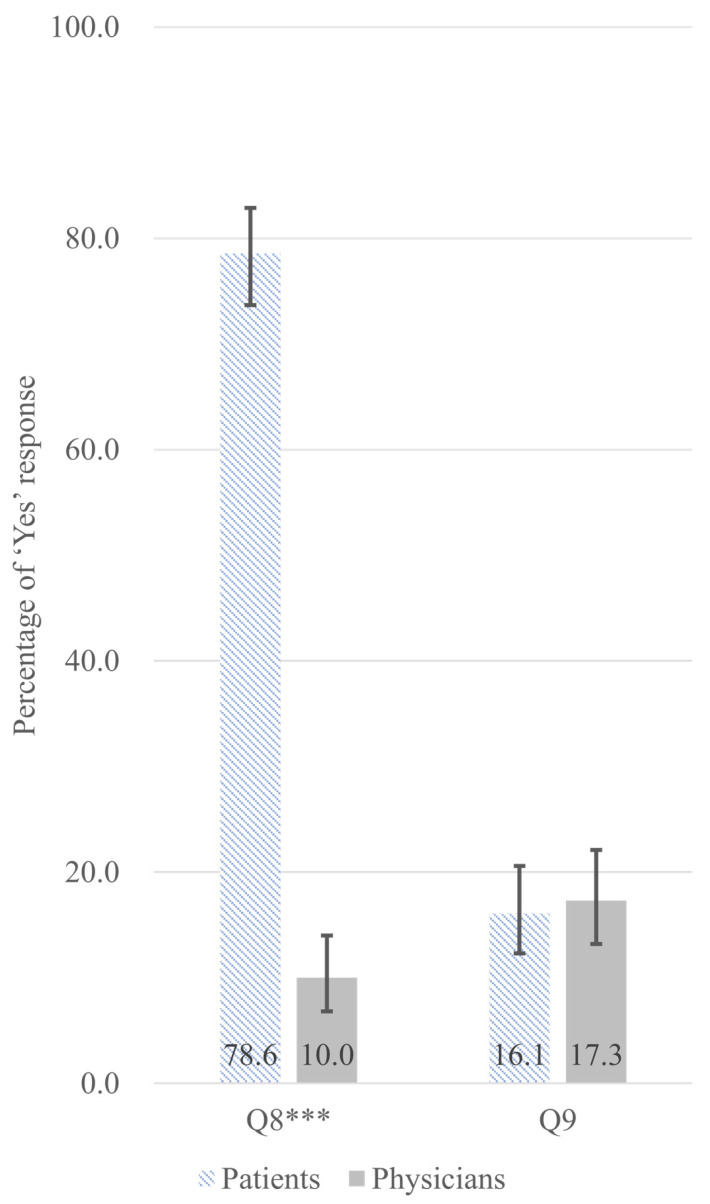
Adjusted predicted probabilities with 95% confidence intervals for ‘Yes’ responses for perceived misuse and abuse of prescription medication (Q8) and awareness of dosage increase since initiation (Q9), comparing patients (hatched bars, *n* = 322) and physicians (gray bars, *n* = 300). Probabilities are adjusted for age and gender using multivariable logistic regression. Error bars indicate 95% confidence intervals. *** *p* < 0.001 (FDR-adjusted *p*-values from Chi-square tests). Survey items. Q8: (for patients) Do you believe that you are currently misusing or abusing your prescribed medication? (for physicians) Do you believe that your patients are currently misusing or abusing their prescribed medications? Q9: (for patients) Are you aware of any dose increase since starting the medication? (for physicians) Are you aware of any dose increase in your patients since starting the medication?

A significant perceptual disparity emerged regarding medical narcotics misuse and abuse. Q8 assessed patient’s self-assessment of their medication-taking behavior. A striking 78.6% of patients self-identified their own current use as potential misuse or abuse, whereas only 10.0% of prescribing physicians suspected their patients might be misusing or abusing these substances (difference 68.6 pp; FDR-adjusted *p* < 0.001) (Figure 2). Complete survey questionnaires are provided in Supplementary Materials S1 (Patient Survey) and Supplementary Materials S2 (Physician Survey). Details explanations of survey items questions are provided in the Appendix A. Response options were limited to Yes/No; no definition of ‘misuse and abuse’ was provided to respondents.

### 3.3. Multivariable Analysis

To account for the differences in age and gender between the two groups, we performed a multivariable logistic regression analysis, with variance inflation factors (VIF) confirming no multicollinearity (all VIFs < 1.4) (Table 3). After adjustment, physicians retained substantially higher odds of awareness across narcotics knowledge items: 14.22-fold for distinguishing medical narcotics from illicit drugs (Q1; 95% CI: 8.62–23.48), 8.50-fold for awareness that prescribed medications are classified as medical narcotics (Q2; 95% CI: 5.21–13.86), and 7.61-fold for awareness of NIMS reporting (Q3; 95% CI: 5.03–11.51). Similarly, physicians showed elevated odds of awareness for narcotics control system accessibility items (Q4–Q7), though effect sizes were smaller (adjusted ORs ranging 1.55–3.87).

The most striking finding was the perceptions of misuse and abuse of prescription medication (Q8): patients had 25-fold higher odds of perceiving their medication use as misuse and abuse compared to physicians (adjusted OR = 0.04 [physicians vs. patients], 95% CI: 0.02–0.06, FDR-adjusted *p* < 0.001). Predicted probabilities showed 77.6% of patients versus 12.8% of physicians endorsed this concern–a 64.8 pp gap.

### 3.4. Subgroup and Interaction Analyses

**Figure 3:**
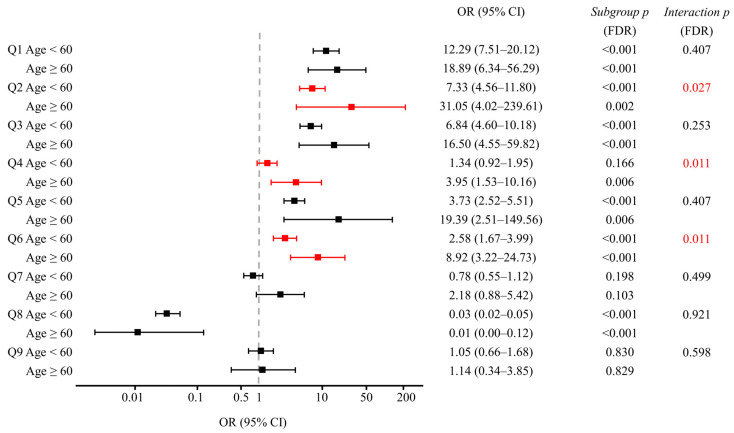
Forest plots of adjusted OR with 95% CIs comparing physicians to patients for survey items Q1–Q9, stratified by age (<60 vs. ≥60 years). Red symbols highlight items with significant group-by-age interactions (FDR-adjusted *p* < 0.05) for Q2, Q4, and Q6. Subgroup *p*-values indicate the significance of physician-patient differences within each age stratum; interaction *p*-values test whether these differences vary by age group. All *p*-values were adjusted for multiple comparisons using the FDR method. The ≥60 physician stratum (*n* = 24) showed wider CIs, warranting cautious interpretation due to the smaller sample size. CI: confidence interval; FDR: false discovery rate; OR: odds ratio. Survey items. Q1: Can you distinguish medical narcotics from illicit drugs? Q2: Are you aware that your prescribed medications are classified as medical narcotics? Q3: Are you aware of the Narcotics Information Management System (NIMS) reporting? Q4: Are you aware of the system for checking a patient’s narcotics prescription history (narcotics prescription status inquiry system)? Q5: Are you aware of physicians’ right to refuse a prescription? Q6: Are you aware of the NIMS Data Service? Q7: Are you willing to try the NIMS Data Service? Q8: (for patients) Do you believe that you are currently misusing or abusing your prescribed medication? (for physicians) Do you believe that your patients are currently misusing or abusing their prescribed medications? Q9: (for patients) Are you aware of any dose increase since starting the medication? (for physicians) Are you aware of any dose increase in your patients since starting the medication?

Subgroup analyses stratified by gender and age, along with interaction testing, revealed no significant interaction for gender, but a significant interaction between group and age was detected for awareness that prescribed medications are classified as medical narcotics (Q2), the narcotics prescription status inquiry system (Q4), and awareness of the NIMS Data Service (Q6) (Figure 3, Table 4).

Regarding the awareness that prescribed medications are classified as medical narcotics (Q2), age-stratified analysis revealed ORs of 7.33 (95% CI: 4.56–11.80) for physicians <60 years versus 31.05 (95% CI: 4.02–239.61; wide due to small *n*) for those ≥60 years (group-by-age interaction *p* = 0.027, FDR-adjusted) (Figure 3). In ≥60 years, awareness was 42.6% in patients vs. 95.8% in physicians, versus 55.7% vs. 90.2% in <60 years (Table 4).

Regarding the awareness of narcotics prescription status inquiry system (Q4), an age-dependent pattern emerged. In the younger age group, no significant difference was observed in awareness between patients and physicians (29.8% vs. 36.2%; FDR-adjusted *p* = 0.166) (Table 4). Conversely, among older participants, a significant disparity was evident, with only 20.2% of patients vs. 50.0% of physicians demonstrating awareness (29.8 pp difference; FDR-adjusted *p* = 0.004).

For the awareness of the NIMS Data Service (Q6), both age groups showed significant differences, but the magnitude of disparity was greater among older participants (Figure 3). In the younger cohort, 15.8% of patients vs. 32.6% of physicians reported awareness (16.8 pp difference; FDR-adjusted *p* < 0.001). Among older participants, this gap more than doubled, with 11.7% of patients compared to 54.2% of physicians demonstrating awareness (42.5 pp difference; FDR-adjusted *p* < 0.001) (Table 4).

### 3.5. Education-Stratified Analysis of Patient Responses

To examine whether patient educational attainment influenced perceptions of opioid analgesics and psychotropic drugs, we stratified survey responses (Q1–Q9) by four education levels: high school or less (*n* = 58, 18.0%), associate degree (*n* = 70, 21.7%), bachelor’s degree (*n* = 181, 56.2%), and graduate degree (*n* = 13, 4.0%) (Table 5). Significant education-related differences were observed across multiple survey items.

For distinguishing medical narcotics from illicit drugs (Q1), affirmative responses increased markedly with higher education level: 0.0% (high school or less), 1.4% (associate degree), 13.8% (bachelor’s), and 46.2% (graduate degree) (overall *p* < 0.001). Post hoc pairwise comparisons using Bonferroni correction revealed significant differences between most education groups, except for high school or below versus associate degree (*p* > 0.999). Similar patterns emerged for awareness that prescribed medications are classified as medical narcotics (Q2), awareness of the NIMS reporting (Q3), awareness of the narcotics prescription status inquiry system (Q4), and the NIMS Data Service awareness (Q6), all demonstrating overall *p* < 0.001 with stepwise increases across education levels. Conversely, awareness of physician’s right to refuse prescription (Q5), perceived misuse and abuse of prescription medication (Q8), awareness of dosage increase since initiation (Q9) showed no significant association with education level (*p* ≥ 0.05). Detailed results are provided in Supplementary Table S3.

### 3.6. Subgroup Analysis by Treatment Duration, Physician Specialty and Clinical Setting

Subgroup analysis by treatment duration revealed that patient-physician perception gaps persisted uniformly regardless of cumulative exposure to medical narcotics prescriptions (FDR-adjusted, Table 6). However, the magnitude of these perception gaps, as reflected by ORs, were greater in patient groups with shorter treatment durations (<36 months) compared to those with longer treatment durations (>36 months). For distinguishing medical narcotics from illicit drugs (Q1), patient awareness increased from 4.8% (6–12 months) to 6.2% (>12–36 months), 18.7% (>36–60 months), and 13.3% (>60 months), while physician awareness remained stable at 57–61%. The OR peaked at 22.58 in the 12–36 months group (patient 6.2% vs. physician 59.4%, difference 53.2 pp, FDR-adjusted *p* < 0.001). Awareness of the NIMS reporting (Q3) similarly persisted across all groups (ORs 6.29–9.02, all FDR-adjusted *p* < 0.001). Perceived misuse and abuse of prescription medication (Q8) showed remarkable consistency across all treatment subgroups, with patients overwhelmingly perceiving themselves as misusing medication (73–82%) while physicians did not (8–13%), ORs 0.01–0.06 (all FDR-adjusted *p* < 0.001). No consistent treatment duration effects were observed for Q4, Q7, and Q9. All detailed values are available in Supplementary Table S4.

Subgroup analysis by physician specialty, physician affiliation, and detailed results are described in Supplementary Document.

## 4. Discussion

Our study revealed three key findings: patients demonstrated poor awareness of narcotics classification due to South Korea’s uniform regulatory system; knowledge deficits were observed in both groups, with many physicians showing limited familiarity with the narcotics control system, and these gaps were particularly pronounced among older patients; and substantial discrepancies exist between physicians’ and patients’ perceptions of narcotics misuse and abuse; patients perceived a much higher rate of misuse and abuse compared to physicians’ estimates.

First, a significant number of patients in our study were unaware that their prescribed medications were classified as narcotics and had difficulty differentiating medical narcotics from illicit drugs. This appears to be due to the uniform classification of both substances as narcotics under the Narcotics Control Act in Korea. Furthermore, the similar pronunciation of the two terms in Korean (*mayak-ryu* and *mayak*) may contribute to this patient confusion.

Unlike South Korea, many other countries classify medical narcotics based on their potential for addiction, dependence, and abuse, allowing for greater regulatory flexibility [10,11,22]. For instance, the U.S. and United Kingdom employ Schedule I–V classification systems that consider medical utility, abuse risk, and prescription protocols, enabling substances to be reclassified as abuse potential changes [10,11]. In contrast, non-medical narcotics are uniformly categorized and strictly regulated as illicit drugs. Japan employs multiple controlled-substance laws (the Narcotics and Psychotropics Control Act, the Stimulants Control Act, the Opium Control Act, and the Cannabis Control Act) that distinguish medical narcotics from illicit drugs, potentially mitigating the conflation observed under South Korea’s uniform classification [22]. These flexible frameworks allow for responsive reclassification based on emerging public health concerns, unlike South Korea’s uniform regulatory approach that integrates medical narcotics with illicit drugs under a single narcotics control system.

Beyond classification systems, digital monitoring infrastructure also influences patient and physician awareness. Unlike the NIMS, which operates separately from electronic health record (EHR) workflows, Taiwan’s National Health Insurance (NHI) MediCloud System provides nationwide, cloud-based accessed to near real-time prescription histories and has been associated with reduced duplication and high physician uptake [16,23,24]. These international experiences suggest that workflow-integrated digital platforms can enhance appropriate narcotic use.

In 2023, the Korean Association of Psychiatrists called for reforms to the Narcotics Control Act, highlighting the problematic classification that groups psychotropic drugs with conventional narcotics [25]. To mitigate these systemic challenges, the Association has proposed reclassifying psychotropic drugs into a distinct legal category. The current classification leads to patient misunderstandings, where medically necessary treatments are erroneously equated with illicit drugs, which can result in prescription reluctance and the erosion of trust in physicians [9,12]. Ultimately, this confusion can foster conflict and mistrust between patients and physicians, which may diminish patient adherence and complicate treatment [26].

Second, although physicians demonstrated significantly higher awareness than patients across several domains, the level of knowledge regarding the narcotics framework was limited in both groups. Nearly half of prescribing physicians were unable to distinguish between medical narcotics and illicit drugs (41.3%), and 9.3% were unaware that their prescribed medications were classified as narcotics (Table 2). Similarly, many physicians showed limited familiarity with key safety systems: 20.0% were unaware of the NIMS reporting (Q3), 62.7% were unaware of the narcotics prescription status inquiry system (Q4), and 65.7% had no knowledge of the NIMS Data Service (Q6). Notably, while 80.0% of physicians were aware that narcotics prescriptions are reported to the NIMS (Q3), only 34.3% demonstrated knowledge of the specialized NIMS Data Service function (Q6). This discrepancy reveals a superficial understanding of the system’s comprehensive capabilities, indicating that substantial knowledge deficits exist even within the physician group.

Age-stratified analyses revealed that the physician-patient perception gap was particularly marked in the older cohort (Figure 3). A significant group-by-age interaction was identified for awareness that prescribed medications are classified as medical narcotics (Q2), the narcotics prescription status inquiry system (Q4), and the NIMS Data Service (Q6). While physicians aged over 60 years maintained awareness levels comparable to or exceeding those of younger colleagues, corresponding patient awareness was markedly lower. This age-dependent widening likely reflects sustained professional familiarity among older physicians and lower awareness among older patients.

Among older patients, lower digital literacy, limited educational attainment, and historical exposure to anti-illicit-drug campaigns that conflated medical narcotics with illicit drugs may contribute to reduced system awareness [27,28]. Conversely, older physicians may benefit from continuous professional engagement with digital health platforms and firsthand experience of evolving narcotics regulations during their careers [29]. These observations suggest that age-tailored educational interventions may be more effective than uniform approaches. However, because physicians’ digital literacy and system utilization were not directly measured in this survey, and given the cross-sectional design that precludes causal inference, these proposed mechanisms remain hypotheses. Prospective studies incorporating validated eHealth literacy scales, objective EHR access logs, and stratification by practice setting and experience will be needed to test these proposed mechanisms.

Our education-stratified analysis revealed that higher educational attainment was strongly associated with greater awareness of medical narcotics classification, and regulatory systems. The gradient effect was particularly striking for distinguishing medical narcotics from illicit drugs (Q1), suggesting health literacy and formal education play pivotal roles in shaping foundational knowledge. Notably, however, perceived misuse and abuse of prescription medication (Q8) showed no significant association with education level (*p* = 0.430). This finding indicates that patient concerns about medication safety and misuse (78.6% overall) transcend educational background, likely stemming from broader societal factors such as media coverage or cultural stigma.

These findings emphasize the need for a dual-track approach to patient education: simplified, accessible materials tailored to diverse literacy levels for knowledge-based items, and universal communication approaches addressing medication safety concerns that affect patients regardless of educational attainment. Physicians should consider baseline educational status when counseling patients about medical narcotics classification and regulatory systems, while recognizing that concerns about misuse and abuse require broader, education-independent interventions that address societal misconceptions and stigma.

Beyond demographic factors, treatment duration also emerged as a critical determinant of patient awareness. Patient awareness distinguishing medical narcotics from illicit drugs (Q1) remained nearly unchanged from 4.8% (6–12 months) to 6.2% (>12–36 months) during the first 36 months of treatment, with the patient-physician gap reaching its maximum (OR = 22.58) in the >12–36 months group (all *p* < 0.001) (Table 6). These findings identify the first 36 months of treatment as a critical ‘golden window’ for patient education, as early misconceptions are remarkably resistant to correction.

Enhanced outreach of narcotics control system is essential for both patients and physicians. Despite South Korea’s narcotics control system being modeled after the U.S. approach, awareness and understanding of NIMS among physicians and patients remain low [15,16]. Moreover, according to healthcare providers using NIMS, responders reported that they primarily utilized the system for administrative reporting purposes rather than for clinical decision-making [15]. In the U.S., 72.0% of physicians demonstrated awareness of PDMP, and 53.0% reported actual experience [30]. PDMP implementation resulted in 20–30% reduction in opioid analgesics prescriptions and 10–15% decrease in overdose death within 2 years [31,32]. However, in our study, fewer than 40% of Korean physicians were aware of the existence of the narcotics control system (Q4; 37.3%, Q6; 34.3%). This finding suggests that systemic promotion is essential for the effective implementation of the narcotics control system. It is also important to explain medical narcotics prescriptions to patients in detail and obtain their consent. In the U.S., physicians are encouraged to inform patients about the risks and responsibilities associated with chronic opioid analgesics prescriptions and to obtain consent before starting treatment [33]. In Japan, patient explanation and consent before initiating narcotics treatment are recommended [22].

Third, a profound discrepancy emerged: 78.6% of patients self-identified their current use as potential misuse and abuse, whereas only 10.0% of physicians suspected misuse among their patients (Table 2). This gap may reflect differences in reference frames between patients and physicians in South Korea’s stringent regulatory context [34,35]. Perceived misuse and abuse of prescription medication (Q8) demonstrated remarkable consistency across all ten subgroups. Regardless of treatment duration, medical specialty, or healthcare setting, patients consistently perceived themselves as misusing medication (73–82%) while physicians did not (8–13%), with ORs ranging from 0.01 to 0.06 (all *p* < 0.001; Table 6, Supplementary Document). This consistent pattern indicates that self-stigmatization poses a significant barrier to treatment adherence across all patient populations and practice settings. The perceptual gap between patients and physicians should be addressed in clinical practice to improve treatment outcomes and patient-physician trust.

Importantly, these percentages should not be interpreted as comparable prevalence estimates; they indicate a perception gap between self-assessed behavior and clinical suspicion–a recognized limitation. However, this finding highlights a fundamental difference in how patients and physicians define ‘misuse’.

While classical social desirability bias would predict under-reporting of stigmatized behaviors such as medication misuse, the unexpectedly high patient endorsement likely stems from a different mechanism: definitional ambiguity combined with regulatory stigma. Patients, operating within a highly stigmatized and rule-bound system, may interpret ‘misuse’ overly broadly (e.g., any deviation from prescribed timing, breakthrough dosing, or even long-term therapeutic use). Conversely, physicians apply narrower clinical criteria (dose escalation, doctor shopping, diversion).

This definitional mismatch is further reinforced by structural factors. South Korea’s Narcotics Control Act uniformly classifies medical narcotics with illicit drugs, prioritizing population-level safety over individualized clinical judgment, thereby generating patient confusion and overly broad interpretations of ‘misuse’. Future research should employ validated multi-item instruments with clear operational definitions and vignette-based items to differentiate minor non-adherence from clinically significant misuse.

This study identified a significant perceptual gap between patients and physicians regarding narcotic use definitions. To address this gap, several evidence-based approaches warrant consideration. First, developing standardized educational materials–in collaboration with Ministry of Food and Drug Safety and medical societies–could clarify the distinction between therapeutic use and misuse, potentially reducing self-stigmatization [1]. Second, integrating such education into routine clinical practice, supported by appropriate reimbursement mechanisms, may enhance patient-physician communication while minimizing administrative burden. Third, establishing broader societal consensus on narcotic use boundaries would address the definitional ambiguity identified in this study. However, implementation must balance thoroughness with efficiency to avoid deterring necessary treatment [36,37]. International models, such as informed consent practices in the U.S. and patient education guidelines in Japan, offer potential frameworks for culturally adapted approaches [22,33].

Our findings suggest several patterns with international applicability. First, the patient-physician perceptual gap likely reflects universal differences in clinical perspectives. Patients assess medication use through stigma and treatment-related fears, whereas physicians apply evidence-based clinical criteria. Second, low awareness of prescription monitoring tools despite high awareness of mandatory reporting is likely to be observed in systems prioritizing administrative documentation over clinical workflow integration. Taiwan’s NHI MediCloud System (formerly PharmaCloud) demonstrated 2.74-fold increases in physician utilization, which supports this mechanism’s generalizability [38].

Conversely, Korea-specific factors limit direct extrapolation. Korea’s uniform classification of prescribed medications with illicit drugs amplifies patient confusion beyond tiered regulatory systems. The notably high patient-reported misuse rate (78.6%) likely reflects strict regulation combined with high treatment adherence expectations. Additionally, Korea’s direct specialist access without referral gatekeeping creates unique prescribing patterns. Therefore, while the underlying clinical mechanisms (medication stigma, patient education deficit, clinical workflow barriers) are transferable, caution is warranted when applying our findings to different practice environments.

This study has several limitations. First, its reliance on self-reported survey data may introduce social desirability and recall biases, and the non-randomized design prevented investigation of various confounders. Second, this purposive sampling of three high-prescribing specialties limits generalizability; perceptions and workflows in primary care, internal medicine, orthopedics, and emergency settings may differ, so findings are most applicable to chronic, non-cancer outpatient contexts. Future studies should broaden to multidisciplinary and primary care environments to assess heterogeneity and enhance external validity. Although these specialties were chosen because their patients are generally more familiar with medical narcotics, this selection may not represent the general healthcare population. The physician response rate of 38.6% (300/800) may introduce non-response bias; however, demographic comparison with national workforce data suggests reasonable representativeness within the sampled specialties. Lack of non-responder data precluded formal bias assessment or post-stratification weighting. Third, our cross-sectional design captures perceptions at a single time point, precluding temporal or causal inference. A longitudinal cohort study with repeated measures is warranted to evaluate how perceptions change over time and in response to interventions. Fourth, this survey instrument was not adapted from a previously validated scale, and formal pre-study construct validation was not conducted, which may affect the precision of measurement. Future studies should use validated instruments and conduct comprehensive psychometric evaluation. Fifth, our interpretation of the age-related pattern is constrained by the lack of direct measures for physicians’ digital literacy and system utilization, and the potential for selective survivor bias among older physicians who remain active in practice. Sixth, the item ‘Q8’ lacked operational definitions and included only binary response options, limiting construct validity and precluding sensitivity analyses. Future studies should employ include middle-category response options. Finally, the scope was limited to the clinical use of medical narcotics and did not address misuse involving illicit drugs, which was a deliberate decision aligned with the study’s focus on standard medical care.

However, this is the first study to assess the perceptions of medical narcotics and the narcotics control system among patients and physicians in South Korea. Our findings revealed that a substantial percentage of patients did not recognize their prescribed medications were classified as narcotics and had difficulty distinguishing medical narcotics from illicit drugs. We also identified considerable discrepancies in the perceptions of narcotics misuse and abuse between patients and physicians, coupled with a limited understanding of the narcotics control system among both groups. These disparities were particularly pronounced among the elderly population.

## 5. Conclusions

In this study, we found significant perceptual discrepancies regarding opioid analgesics and psychotropic drugs between patients and physicians. Both groups showed limited awareness of opioid analgesics and psychotropic drugs and the narcotics control system, especially those over 60 years of age. Therefore, targeted educational initiatives are crucial to bridge the existing perceptual and knowledge gaps.

## Figures and Tables

**Table 1:** Demographic and clinical characteristics of patient and physician groups.

Variables	Patient (*n* = 322)	Physician (*n* = 300)	*p*-Value
Demographics			
Age (years), mean ± SD	51.25 ± 15.36	49.14 ± 7.34	0.027
Age (group), *n* (%)			<0.001
<60 years	228 (70.8)	276 (92.0)	
≥60 years	94 (29.2)	24 (8.0)	
Gender, *n* (%)			<0.001
Male	116 (36.0)	250 (83.3)	
Female	206 (64.0)	50 (16.7)	
Patient Clinical Characteristics			
Prescription Duration, *n* (%)			
6–12 months	90 (28.0)	N/A	
>12–36 months	123 (38.2)	N/A	
>36–60 months	76 (23.6)	N/A	
>60 months	33 (10.2)	N/A	
Educational Attainment, *n* (%)			
High school or Less	58 (18.0)	N/A	
Associate Degree	70 (21.7)	N/A	
Bachelor’s Degree	181 (56.2)	N/A	
Graduate Degree	13 (4.0)	N/A	
K-eHEALS (score), mean ± SD	26.30 ± 6.43	N/A	
Residence, *n* (%)			
Urban	277 (86.0)	N/A	
Rural	45 (14.0)	N/A	
Primary Indication, *n* (%)			
Sleep disorder	81 (25.2)	N/A	
Anxiety disorder	57 (17.7)	N/A	
Depressive disorder	38 (11.8)	N/A	
Pain	23 (7.1)	N/A	
Other diseases	123 (38.2)	N/A	
Physician Professional Characteristics			
Medical Specialty, *n* (%)			
Psychiatry	N/A	193 (64.4)	
Neurology	N/A	82 (27.3)	
Anesthesiology and Pain Medicine	N/A	25 (8.3)	
Work Setting, *n* (%)			
Private clinic	N/A	131 (43.7)	
University hospital	N/A	124 (41.3)	
Employed physician	N/A	45 (15.0)	
Work Period (year)	N/A	17.63 ± 7.15	
Work Residence, *n* (%)			
Urban	N/A	255 (85.0)	
Rural	N/A	45 (15.0)	

K-eHEALS: The Korean eHealth Literacy Scale. SD: standard deviation. N/A indicates group-specific variables not applicable to the other group. Other diseases include panic disorder, bipolar disorder, obsessive–compulsive disorder, and epilepsy. Data are presented as *n* (%) for categorical variables (percentages calculated using group-specific totals as denominators) and mean ± SD for continuous variables. Statistical comparisons performed using independent *t*-tests for continuous variables and Chi-square tests for categorical variables.

**Table 2:** Comparison of perceptions between patients and physicians (Yes responses only).

Survey Questions		Patients (*n* = 322), *n* (%)	Physicians (*n* = 300) *n* (%)	Patients	Physicians	Proportion Difference (95% CI)	*p*-Value	FDR-Adjusted *p*-Value
				Proportion, % (95% CI)				
Narcotics Knowledge and Awareness	Q1	32 (9.9)	176 (58.7)	9.9 (6.9–13.7)	58.7 (52.9–64.3)	48.8 (42.3–55.2)	<0.001	<0.001
	Q2	167 (51.9)	272 (90.7)	51.9 (46.3–57.4)	90.7 (86.8–93.7)	38.8 (32.4–45.2)	<0.001	<0.001
	Q3	110 (34.2)	240 (80.0)	34.2 (29.0–39.6)	80.0 (75.0–84.4)	45.8 (39.0–52.7)	<0.001	<0.001
Narcotics Control System Accessibility	Q4	87 (27.0)	112 (37.3)	27.0 (22.2–32.2)	37.3 (31.8–43.1)	10.3 (3.0–17.6)	0.006	0.008
	Q5	168 (52.2)	243 (81.0)	52.2 (46.6–57.7)	81.0 (76.1–85.3)	28.8 (21.8–35.9)	<0.001	<0.001
	Q6	47 (14.6)	103 (34.3)	14.6 (10.9–18.9)	34.3 (29.0–40.0)	19.7 (13.1–26.4)	<0.001	<0.001
	Q7	135 (41.9)	120 (40.0)	41.9 (36.5–47.5)	40.0 (34.4–45.8)	1.9 (−5.8–9.7)	0.626	0.692
Misuse and Abuse	Q8	253 (78.6)	30 (10.0)	78.6 (73.7–82.9)	10.0 (6.8–14.0)	68.6 (62.9–74.2)	<0.001	<0.001
	Q9	52 (16.1)	52 (17.3)	16.1 (12.3–20.6)	17.3 (13.2–22.1)	1.2 (−4.7–7.1)	0.692	0.692

Values are counts and percentages of ‘Yes’ responses; No responses omitted. Percentages calculated using group-specific denominators. CI: Confidence Interval; FDR: False Discovery Rate. Chi-square tests (Fisher’s exact test when appropriate) used for comparisons. Q1: Distinguishing medical narcotics from illicit drugs; Q2: Awareness that prescribed medications are classified as medical narcotics; Q3: Awareness of the Narcotics Information Management System (NIMS) reporting; Q4: Awareness of the narcotics prescription status inquiry system; Q5: Awareness of physician’s right to refuse prescription; Q6: Awareness of the NIMS Data Service; Q7: Willingness to try the NIMS Data Service; Q8: Perceived misuse and abuse of prescription medication; Q9: Awareness of dosage increase since initiation.

**Table 3:** Age and gender adjusted logistic regression analysis of perceptual differences (physicians vs. patients as reference).

Variables		Univariate OR (95% CI)	FDR-Adjusted *p*-Value	Adjusted OR (95% CI)	FDR-Adjusted *p*-Value	Predicted Probability (95% CI)		Risk Difference (95% CI)	Hosmer–Lemeshow Goodness-of-Fit Test *p*-Value (FDR-Adjusted)
						Patient	Physician		
Narcotics Knowledge and Awareness	Q1	12.86 (8.36–19.80)	<0.001	14.22 (8.62–23.48)	<0.001	9.6 (6.3–12.9)	59.4 (53.5–65.4)	49.8 (42.9–56.7)	0.936
	Q2	9.02 (5.77–14.09)	<0.001	8.50 (5.21–13.86)	<0.001	52.1 (46.5–57.7)	89.7 (85.3–94.1)	37.7 (30.2–45.1)	0.105
	Q3	7.71 (5.35–11.10)	<0.001	7.61 (5.03–11.51)	<0.001	34.4 (29.1–39.7)	78.8 (73.5–84.0)	44.4 (36.5–52.2)	0.768
Narcotics Control System Accessibility	Q4	1.61 (1.15–2.26)	0.008	1.55 (1.05–2.28)	0.034	27.0 (22.0–32.0)	36.7 (30.6–42.9)	9.7 (1.3–18.1)	0.007
	Q5	3.91 (2.72–5.61)	<0.001	3.87 (2.57–5.80)	<0.001	52.3 (46.7–57.8)	80.7 (75.5–85.8)	28.4 (20.4–36.4)	0.936
	Q6	3.06 (2.09–4.52)	<0.001	3.06 (1.96–4.78)	<0.001	14.5 (10.6–18.5)	34.3 (28.4–40.2)	19.8 (12.3–27.3)	0.647
	Q7	0.92 (0.67–1.27)	0.693	0.95 (0.66–1.37)	0.768	41.9 (36.4–47.4)	40.6 (34.3–46.8)	1.3 (−7.4–10.1)	0.208
Misuse and Abuse	Q8	0.03 (0.02–0.05)	<0.001	0.04 (0.02–0.06)	<0.001	77.6 (73.0–82.2)	12.8 (8.3–17.2)	64.9 (57.8–71.9)	0.743
	Q9	1.09 (0.71–1.66)	0.693	1.62 (0.98–2.66)	0.065	14.5 (10.7–18.2)	21.3 (16.4–26.2)	6.8 (0.7–12.9)	0.943

CI: Confidence Interval; FDR: False Discovery Rate; OR: Odds Ratio. All logistic regression models adjusted for age and gender. Odds ratios compare physicians to patients, with patients as the reference group (OR >1 indicates higher odds in physicians). Predicted probabilities represent estimated proportions of ‘Yes’ responses for each group based on the adjusted model. Risk difference calculated as (physicians-patients). Hosmer-Lemeshow test assesses model calibration (*p* > 0.05 indicates acceptable goodness-of-fit). All *p*-values were adjusted for multiple comparisons using the FDR method. Q1: Distinguishing medical narcotics from illicit drugs; Q2: Awareness that prescribed medications are classified as medical narcotics; Q3: Awareness of the Narcotics Information Management System (NIMS) reporting; Q4: Awareness of the narcotics prescription status inquiry system; Q5: Awareness of physician’s right to refuse prescription; Q6: Awareness of the NIMS Data Service; Q7: Willingness to try the NIMS Data Service; Q8: Perceived misuse and abuse of prescription medication; Q9: Awareness of dosage increase since initiation.

**Table 4:** Age-stratified analysis of survey responses (Yes responses only).

Survey Questions	Patient <60 (*n* = 228)	Physician <60 (*n* = 276)	FDR-Adjusted *p*-Value	Patient ≥60 (*n* = 92)	Physician ≥60 (*n* = 24)	FDR-Adjusted *p*-Value
Narcotics Knowledge and Awareness, *n* (%)						
Q1: Distinguish medical narcotics from illicit drugs	23 (10.1)	160 (58.0)	<0.001	9 (9.6)	16 (66.7)	<0.001
Q2: Awareness of prescribed medications as classified as medical narcotics	127 (55.7)	249 (90.2)	<0.001	40 (42.6)	23 (95.8)	<0.001
Q3: Awareness of the NIMS reporting	82 (36.0)	219 (79.3)	<0.001	28 (29.8)	21 (87.5)	<0.001
Narcotics Control System Accessibility, *n* (%)						
Q4: Awareness of the narcotics prescription status inquiry system	68 (29.8)	100 (36.2)	0.166	19 (20.2)	12 (50.0)	0.004
Q5: Awareness of physician’s right to refuse prescription	117 (51.3)	220 (79.7)	<0.001	51 (54.3)	23 (95.8)	<0.001
Q6: Awareness of the NIMS Data Service	36 (15.8)	90 (32.6)	<0.001	11 (11.7)	13 (54.2)	<0.001
Q7: Willingness to try the NIMS Data Service	102 (44.7)	107 (38.8)	0.198	33 (35.1)	13 (54.2)	0.098
Misuse and Abuse, *n* (%)						
Q8: Perceived misuse and abuse of prescription medication	183 (80.3)	29 (10.5)	<0.001	70 (74.5)	1 (4.2)	<0.001
Q9: Awareness of dosage increase since initiation	38 (16.7)	48 (17.4)	0.830	14 (14.9)	4 (16.7)	0.760

Values are counts and percentages of ‘Yes’ responses; No responses omitted. Data presented as *n* (%). Percentages calculated using group-specific denominators. FDR: false discovery rate; NIMS: Narcotics Information Management System. Chi-square tests used for comparisons with each age stratum. All *p*-values were adjusted for multiple comparisons using the FDR method. Group-by-age interaction *p*-values (FDR-adjusted) shown in Figure 3. The ≥60 physician stratum (*n* = 24) showed wider CIs, warranting cautious interpretation due to the smaller sample size. Expanded version: Supplementary Table S2.

**Table 5:** Correlation analysis with Patient’s Education (Yes responses only).

Survey Questions		Education				Overall *p*-Value	Post Hoc Test
		High School or Less	Associate Degree	Bachelor’s Degree	Graduate Degree		
Narcotics Knowledge and Awareness, *n* (%)	Q1: 32 (9.9)	0 (0.0)	1 (1.4)	25 (13.8)	6 (46.2)	<0.001	0.017
	Q2: 167 (51.9)	7 (12.1)	22 (31.4)	126 (69.6)	12 (92.3)	<0.001	<0.001
	Q3: 110 (34.2)	6 (10.3)	15 (21.4)	79 (43.6)	10 (76.9)	<0.001	<0.001
Narcotics Control System Accessibility, *n* (%)	Q4: 87 (27.0)	5 (8.6)	13 (18.6)	64 (35.4)	5 (38.5)	<0.001	<0.001
	Q5: 168 (52.2)	28 (48.3)	28 (40.0)	105 (58.0)	7 (53.8)	0.072	>0.999
	Q6: 47 (14.6)	2 (3.4)	4 (5.7)	36 (19.9)	5 (38.5)	<0.001	0.017
	Q7: 135 (41.9)	18 (31.0)	21 (30.0)	91 (50.3)	5 (38.5)	0.007	0.063
Misuse and Abuse, *n* (%)	Q8: 253 (78.6)	45 (77.6)	60 (85.7)	138 (76.2)	10 (76.9)	0.430	>0.999
	Q9: 52 (16.1)	11 (19.0)	6 (8.6)	34 (18.8)	1 (7.7)	0.178	>0.999

Values are counts and percentages of ‘Yes’ responses; No responses omitted. Total patients *n* = 322. Data presented as *n* (%). Post hoc test: Post hoc pairwise comparisons (Bonferroni-adjusted) of ‘high school or less vs. bachelor’s degree’. Chi-square tests (Fisher’s exact test when appropriate) used for comparisons. Q1: Distinguishing medical narcotics from illicit drugs. Q2: Awareness that prescribed medications are classified as medical narcotics. Q3: Awareness of the NIMS reporting. Q4: Awareness of the narcotics prescription status inquiry system. Q5: Awareness of physician’s right to refuse prescription. Q6: Awareness of the NIMS Data Service. Q7: Willingness to try the NIMS Data Service. Q8: Perceived misuse and abuse of prescription medication. Q9: Awareness of dosage increase since initiation.

**Table 6:** Subgroup analysis by patient treatment duration: Multivariable logistic regression.

Category	Subgroup	Outcome	OR (95% CI)	FDR-Adjusted *p*-Value
Patient	Patient:	Q1	20.41 (7.74–53.84)	<0.001
Subgroup	Treatment duration	Q2	6.15 (2.83–13.36)	<0.001
	6–12 months	Q3	6.29 (3.19–12.41)	<0.001
	(*n* = 90)	Q4	1.00 (0.53–1.88)	0.995
		Q5	3.57 (1.83–6.94)	<0.001
		Q6	2.70 (1.25–5.83)	0.018
		Q7	1.13 (0.61–2.10)	0.775
		Q8	0.04 (0.02–0.09)	<0.001
		Q9	1.22 (0.55–2.70)	0.775
	Patient:	Q1	22.58 (10.06–50.65)	<0.001
	Treatment duration	Q2	9.33 (5.18–16.81)	<0.001
	>12–36 months	Q3	9.02 (5.27–15.47)	<0.001
	(*n* = 123)	Q4	1.36 (0.82–2.27)	0.259
		Q5	3.10 (1.86–5.18)	<0.001
		Q6	3.76 (1.97–7.17)	<0.001
		Q7	1.00 (0.61–1.61)	0.985
		Q8	0.03 (0.01–0.05)	<0.001
		Q9	1.60 (0.84–3.02)	0.195
	Patient:	Q1	6.63 (3.06–14.35)	<0.001
	Treatment duration	Q2	8.36 (3.94–17.75)	<0.001
	>36–60 months	Q3	6.85 (3.43–13.66)	<0.001
	(*n* = 76)	Q4	2.03 (0.98–4.19)	0.071
		Q5	4.60 (2.32–9.12)	<0.001
		Q6	2.39 (1.10–5.19)	0.041
		Q7	0.69 (0.37–1.31)	0.295
		Q8	0.03 (0.01–0.08)	<0.001
		Q9	1.07 (0.47–2.42)	0.875
	Patient:	Q1	9.34 (2.94–29.66)	<0.001
	Treatment duration	Q2	21.42 (5.99–76.63)	<0.001
	>60 months	Q3	6.37 (2.20–18.42)	0.001
	(*n* = 33)	Q4	3.82 (1.15–12.65)	0.037
		Q5	3.54 (1.22–10.29)	0.030
		Q6	4.09 (1.27–13.19)	0.030
		Q7	0.78 (0.29–2.07)	0.619
		Q8	0.06 (0.02–0.21)	<0.001
		Q9	7.91 (0.88–71.42)	0.074

Multivariable logistic regression models were adjusted for age and gender. All *p*-values were adjusted for multiple comparisons using the FDR method. Patient subgroup analysis included all physician participants (*n* = 300). CI, confidence interval; FDR: false discovery rate; OR, odds ratio. Q1: Distinguishing medical narcotics from illicit drugs. Q2: Awareness that prescribed medications are classified as medical narcotics. Q3: Awareness of the NIMS reporting. Q4: Awareness of the narcotics prescription status inquiry system. Q5: Awareness of physician’s right to refuse prescription. Q6: Awareness of the NIMS Data Service. Q7: Willingness to try the NIMS Data Service. Q8: Perceived misuse and abuse of prescription medication. Q9: Awareness of dosage increase since initiation.

## Data Availability

The datasets used and/or analyzed in the current study are available from the corresponding author on reasonable request.

## Supplementary Materials

The following supporting information can be downloaded at: https://www.mdpi.com/article/10.3390/jcm14217734/s1, Supplementary Materials S1: Patient Survey Questions; Supplementary Materials S2: Physician Survey Questions; Supplementary Materials S3: strobe checklist; Supplementary Table S1: Internal Consistency and Corrected item-total correlations for questionnaire table; Supplementary Table S2: Expanded version of Table 4; Supplementary Table S3: Expanded version of Table 5; Supplementary Table S4: Expanded version of Table 6; Supplementary Document: Subgroup analysis documents.

## Abbreviations

The following abbreviations are used in this manuscript:

EHR	Electronic Health Record
FDR	False Discovery Rate
K-eHEALS	Korean version of the eHealth Literacy Scale
NHI	National Health Insurance
NIMS	Narcotics Information Management System
PDMP	Prescription Drug Monitoring Program

## Appendix A. Survey Question Definitions

Q3. NIMS reporting: Mandatory, non-real-time automated submission of all narcotics handling records to national regulatory oversight system.

Q6. NIMS Data Service: Optional, point-of-care clinical lookup tool enabling physicians to actively query a patient’s prescription history.
