# Supplementary material for: Perceptual Discrepancies of Opioid Analgesics and Psychotropic Drugs: A Cross-Sectional Study of Korean Patients and Physicians
Source: J Clin Med. 2025 Oct 31;14(21):7734. doi: 10.3390/jcm14217734 (PMC12609944; doi:10.3390/jcm14217734)
Supplement: Supplementary file 1 [file jcm-14-07734-s001.zip › Supplementary_Document.pdf]

1. *Results*

1.1. Subgroup analysis by Physician Specialty

Subgroup analysis by physician specialty (Neurology, Psychiatry, Anesthesiology and Pain Medicine; Bonferroni-corrected $\alpha = 0.0019$) demonstrated that perception gaps were independent of specialty training (Supplementary Document Table 1). For distinguishing medical narcotics from illicit drugs (Q1), Psychiatry physicians showed the highest physician-patient gap (OR 16.51, 95% CI: 9.52–28.61; patient 9.7% vs. physician 63.3%, difference 53.7pp; $p < 0.001$), followed by Neurology (OR 11.18) and Anesthesiology and Pain Medicine (OR 9.50; all $p < 0.001$). Awareness that prescribed medications are classified as medical narcotics (Q2) was similarly low among patients in Neurology and Psychiatry, with consistent physician-patient gaps (ORs 8.07–9.29; both $p \leq 0.001$). Awareness of NIMS reporting (Q3) was uniformly low among patients across all specialties (33.7–34.6%), with consistent physician awareness (76.9–80.2%; ORs 6.13–8.17; all $p < 0.001$). Awareness of NIMS Data Service (Q6) revealed notable specialty differences, with Anesthesiology and Pain Medicine demonstrating the largest gap (OR 6.36, 95% CI: 2.62–15.44; difference 36.6pp; $p < 0.001$), compared to Neurology (OR 3.17) and Psychiatry (OR 2.76; all $p < 0.001$). Perceived misuse and abuse of prescription medication (Q8) was remarkably consistent across all specialties, with 77.0–77.6% of patients perceiving themselves as misusing medications versus 8.4–13.7% of physicians (ORs 0.01–0.04; all $p < 0.001$). The largest gap was observed among Anesthesiology and Pain Medicine patients (69.1pp). No consistent specialty effects were observed for Q4, Q7, or Q9.

1.2. Subgroup analysis by Hospital Affiliation

Subgroup analysis by physician affiliation (private clinic, employed physician, university hospital; Bonferroni-corrected $\alpha = 0.0019$) demonstrated that perception gaps were independent of practice setting (Supplementary Document Table 1). For distinguishing medical narcotics from illicit drugs (Q1), all affiliations showed large gaps (ORs 9.91–18.58; all $p < 0.001$), with private practice highest. Awareness that prescribed medications are classified as medical narcotics (Q2) and awareness of NIMS reporting (Q3) was uniformly low among patients across all settings (ORs 6.37–11.41 and 7.15–8.89, respectively; all $p < 0.001$). Awareness of NIMS Data Service (Q6) was significant only in private practice (OR 5.57; $p < 0.001$), but not in employed physicians ($p = 0.053$) or university hospitals ($p = 0.178$). Perceived misuse and abuse of prescription medication (Q8) was consistent across all settings, with patients perceiving themselves as misusing medications (73–81%) versus physicians (10–12%; ORs 0.03–0.05; all $p < 0.001$). The largest gap was observed in private practice patients (67.6pp). Awareness of the narcotic prescription status inquiry system (Q4) was significant only in private practice (OR 2.42; $p < 0.001$). No consistent affiliation effects were observed for Q7 or Q9.

2. *Discussion*

Subgroup analysis revealed that patient-physician perception gaps were largely independent of both specialty training and practice setting, suggesting that identified knowledge deficits reflect systemic issues within South Korea's narcotics control framework.

2.1. Physician specialty findings

Across all three specialties, patient awareness remained consistently low for fundamental concepts: distinguishing medical narcotics from illicit drugs (Q1; 9.6–9.7% across specialties) and awareness of NIMS reporting (Q3; 33.7–34.6%). This consistency suggests the problem stems from structural factors–South Korea’s uniform classification system that conflates medical narcotics with illicit drugs.

Anesthesiology and Pain Medicine demonstrated the largest gap in NIMS Data Service awareness (Q6, OR 6.36; 36.6pp difference). This likely reflects chronic pain patients’ more frequent long-term

medical narcotics exposure and pain specialists' higher system engagement. However, even in this specialty, only 51.3% of physicians versus 14.7% of patients were aware of this function.

Self-stigmatization (Q8) was remarkably consistent across specialties, with approximately 77% of patients perceiving themselves as misusing medications versus 8.4–13.7% of physicians. The largest gap among Anesthesiology patients (69.1pp) may reflect heightened societal suspicion regarding chronic pain management.

2.2. Practice setting findings

Analysis by affiliation revealed similar universal gaps for Q1, Q2, Q3, and Q8, confirming that institutional setting does not substantially modify patient awareness. This consistency persisted despite significant differences in patient populations, resources, and practice patterns across private clinics, employed physician practices, and university hospitals.

However, awareness of narcotics control system accessibility (Q4, Q6) was significant only in private practice, suggesting that physicians in private settings demonstrate greater system engagement, potentially due to increased autonomy in clinical decision-making and direct regulatory responsibility.

The independence of perception gaps from specialty and setting indicates that interventions should be universal. Standardized educational protocols can be implemented across all specialties and settings. The private practice findings suggest that system-integrated interventions may be particularly effective where physicians already demonstrate higher monitoring tool engagement, potentially serving as pilots for broader implementation.

Supplementary Document Table S1. Subgroup analysis of patient-physician perception gaps by physician characteristics: Multivariable logistic regression

Category	Subgroup	Outcome	OR (95% CI)	*p*-value	Predicted Probability (95% CI)		Risk Difference (95% CI)	Hosmer-Lemeshow Test (goodness-of-fit)
					Patient	Physician		
Physician Subgroup by Specialty	Neurology (*n* = 82)	Q1	11.18 (5.79–21.60)	<0.001	9.6 (6.3–12.9)	53.1 (42.1–64.1)	43.5 (31.9–55.1)	0.638
		Q2	9.29 (4.04–21.37)	<0.001	52.0 (46.4–57.6)	89.0 (81.9–96.1)	37.0 (27.5–46.6)	0.21
		Q3	8.17 (4.35–15.32)	<0.001	33.7 (28.5–38.9)	79.3 (69.7–88.9)	45.6 (34.3–56.8)	0.124
		Q4	1.66 (0.94–2.91)	0.088	26.4 (21.6–31.3)	36.7 (26.0–47.3)	10.2 (-1.7–22.2)	0.008
		Q5	4.46 (2.33–8.53)	<0.001	52.3 (46.7–57.9)	83.0 (74.3–91.6)	30.7 (20.0–41.3)	0.178
		Q6	3.17 (1.71–5.87)	<0.001	14.5 (10.5–18.4)	34.3 (23.8–44.8)	19.8 (8.4–31.3)	0.355
		Q7	1.43 (0.84–2.42)	0.188	41.6 (36.1–47.1)	50.3 (39.1–61.6)	8.7 (-4.2–21.6)	0.1
		Q8	0.04 (0.02–0.08)	<0.001	77.0 (72.3–81.8)	13.0 (6.0–20.0)	64.0 (54.8–73.3)	0.591
		Q9	2.08 (1.02–4.25)	0.058	14.4 (10.7–18.1)	23.2 (14.3–32.0)	8.7 (-0.7–18.2)	0.36
	Psychiatry (*n* = 193)	Q1	16.51 (9.52–28.61)	<0.001	9.7 (6.4–13.0)	63.3 (56.2–70.5)	53.7 (45.6–61.7)	0.97
		Q2	8.07 (4.60–14.17)	<0.001	52.0 (46.4–57.6)	88.7 (83.2–94.2)	36.7 (28.4–45.0)	0.012
		Q3	7.85 (4.89–12.62)	<0.001	34.6 (29.3–39.8)	78.8 (72.6–85.0)	44.2 (35.6–52.8)	0.667
		Q4	1.49 (0.96–2.29)	0.094	27.1 (22.0–32.1)	35.8 (28.4–43.3)	8.8 (-0.7–18.3)	0.006
		Q5	3.70 (2.34–5.86)	<0.001	52.1 (46.5–57.6)	80.1 (73.7–86.4)	28.0 (19.1–36.9)	0.487
		Q6	2.76 (1.68–4.54)	<0.001	14.6 (10.6–18.5)	32.1 (25.0–39.1)	17.5 (9.1–26.0)	0.16
		Q7	0.80 (0.52–1.21)	0.318	41.7 (36.3–47.2)	36.3 (28.9–43.8)	5.4 (-4.2–15.1)	0.268
		Q8	0.04 (0.02–0.07)	<0.001	77.6 (73.0–82.3)	13.7 (8.2–19.3)	63.9 (56.0–71.8)	0.61
		Q9	1.25 (0.70–2.22)	0.452	14.4 (10.6–18.3)	18.4 (12.7–24.1)	4.0 (-3.0–11.0)	0.156
	Anesthesiology and Pain Medicine (*n* = 25)	Q1	9.50 (3.71–24.30)	<0.001	9.6 (6.3–12.9)	48.5 (28.8–68.2)	38.9 (18.9–58.9)	0.518
		Q2	9.52 (2.17–41.67)	0.005	52.1 (46.6–57.7)	86.0 (75.4–96.7)	33.9 (21.5–46.3)	0.009
		Q3	6.13 (2.32–16.23)	<0.001	34.2 (29.1–39.4)	75.7 (58.4–93.0)	41.5 (23.3–59.7)	0.477
		Q4	2.15 (0.91–5.09)	0.091	26.6 (21.8–31.5)	42.1 (22.0–62.3)	15.5 (-5.4–36.4)	0.161
		Q5	4.98 (1.64–15.10)	0.007	52.0 (46.3–57.6)	84.3 (69.7–98.9)	32.3 (16.4–48.2)	0.25
		Q6	6.36 (2.62–15.44)	<0.001	14.7 (10.7–18.7)	51.3 (31.4–71.1)	36.6 (16.2–56.9)	0.185
		Q7	0.89 (0.38–2.09)	0.784	41.9 (36.3–47.4)	38.7 (19.1–58.4)	3.1 (-17.6–23.8)	0.408
		Q8	0.01 (0.00–0.10)	<0.001	77.5 (72.7–82.3)	8.4 (-0.6–17.3)	69.1 (58.2–80.1)	0.406
		Q9	4.08 (1.52–10.98)	0.007	14.4 (10.7–18.1)	34.9 (16.3–53.5)	20.5 (1.6–39.4)	0.219
Physician Subgroup by Affiliation	Private Clinic (*n* = 131)	Q1	18.58 (9.93–34.75)	<0.001	9.6 (6.3–12.8)	63.0 (54.3–71.6)	53.4 (44.1–62.7)	0.412
		Q2	11.41 (5.43–23.96)	<0.001	52.5 (46.9–58.1)	89.6 (83.9–95.3)	37.1 (28.7–45.5)	0.712
		Q3	8.89 (5.14–15.37)	<0.001	34.1 (28.9–39.4)	80.5 (73.2–87.7)	46.3 (37.0–55.7)	0.498
		Q4	2.42 (1.51–3.89)	<0.001	26.9 (21.9–31.8)	46.9 (37.9–56.0)	20.1 (9.3–30.8)	0.004
		Q5	4.90 (2.81–8.54)	<0.001	52.1 (46.5–57.7)	84.0 (77.2–90.9)	31.9 (22.6–41.2)	0.075
		Q6	5.57 (3.32–9.36)	<0.001	14.8 (10.7–18.9)	50.3 (41.4–59.3)	35.5 (25.3–45.7)	0.266

	Q7	1.01 (0.64–1.60)	0.975	41.4 (35.9–46.9)	41.6 (32.6–50.6)	0.2 (-10.7–11.1)	0.065
	Q8	0.03 (0.01–0.06)	<0.001	77.9 (73.2–82.6)	10.3 (4.3–16.3)	67.6 (59.3–75.9)	0.278
	Q9	1.41 (0.74–2.68)	0.339	14.4 (10.6–18.2)	19.4 (12.7–26.1)	5.1 (-2.6–12.7)	0.315
Employed Physician	Q1	9.91 (4.64–21.15)	<0.001	9.7 (6.3–13.0)	51.5 (36.8–66.2)	41.8 (26.6–57.0)	0.401
(*n* = 45)	Q2	8.87 (3.04–25.87)	<0.001	51.8 (46.2–57.3)	87.9 (78.5–97.4)	36.2 (24.6–47.7)	0.018
	Q3	7.17 (3.30–15.55)	<0.001	33.9 (28.6–39.1)	76.2 (63.7–88.6)	42.3 (28.5–56.1)	0.089
	Q4	1.46 (0.72–2.96)	0.327	26.3 (21.5–31.1)	33.3 (19.6–47.0)	7.0 (-7.7–21.7)	0.064
	Q5	3.41 (1.59–7.32)	0.003	51.7 (46.0–57.3)	79.3 (66.5–92.1)	27.6 (13.3–42.0)	0.829
	Q6	2.21 (0.99–4.93)	0.080	14.3 (10.5–18.2)	24.8 (12.2–37.4)	10.4 (-2.8–23.7)	0.366
	Q7	0.85 (0.43–1.66)	0.625	41.6 (36.1–47.2)	37.8 (23.4–52.1)	3.9 (-11.8–19.6)	0.15
	Q8	0.04 (0.02–0.11)	<0.001	77.1 (72.3–81.9)	13.8 (4.4–23.2)	63.3 (52.2–74.5)	0.288
	Q9	1.93 (0.82–4.55)	0.173	14.4 (10.6–18.1)	23.7 (11.9–35.5)	9.3 (-3.0–21.6)	0.272
University Hospital	Q1	12.92 (7.15–23.35)	<0.001	9.7 (6.4–13.1)	58.5 (49.5–67.4)	48.7 (39.0–58.5)	0.083
(*n* = 124)	Q2	6.37 (3.42–11.86)	<0.001	52.0 (46.4–57.6)	85.8 (78.8–92.9)	33.9 (24.4–43.4)	0.057
	Q3	7.15 (4.17–12.24)	<0.001	34.6 (29.3–39.8)	77.2 (69.5–84.9)	42.6 (32.9–52.3)	0.346
	Q4	1.00 (0.60–1.67)	0.988	26.8 (21.9–31.8)	27.1 (18.7–35.4)	0.2 (-9.9–10.3)	0.003
	Q5	3.39 (2.00–5.72)	<0.001	52.4 (46.8–58.0)	78.7 (70.9–86.4)	26.2 (16.2–36.2)	0.978
	Q6	1.51 (0.83–2.76)	0.229	14.5 (10.6–18.5)	20.2 (12.7–27.7)	5.7 (-3.1–14.4)	0.177
	Q7	0.90 (0.56–1.44)	0.737	42.2 (36.7–47.7)	39.4 (30.1–48.7)	2.8 (-8.5–14.1)	0.413
	Q8	0.05 (0.03–0.09)	<0.001	77.2 (72.5–81.9)	15.5 (8.9–22.1)	61.7 (52.8–70.5)	0.573
	Q9	1.97 (1.04–3.73)	0.058	14.5 (10.8–18.2)	22.6 (15.4–29.8)	8.1 (0.1–16.0)	0.602

Multivariable logistic regression models were adjusted for age and gender. Statistical significance was determined using Bonferroni-corrected $\alpha = 0.0019$ to account for multiple comparisons across nine outcomes. OR, odds ratio; CI, confidence interval; NIMS: Narcotics Information Management System. Predicted probabilities represent model-based estimates for patients and physician groups. Risk difference indicates the percentage point difference between physician and patient awareness. Hosemer-Lemeshow test assesses model goodness-of-fit, with $p > 0.05$ indicating adequate fit. Physician subgroup analysis included all patient participants (*n* = 332).

Q1: Distinguishing medical narcotics from illicit drugs; Q2: Awareness that prescribed medications are classified as medical narcotics; Q3: Awareness of the NIMS reporting; Q4: Awareness of the narcotics prescription status inquiry system; Q5: Awareness of physician's right to refuse prescription; Q6: Awareness of the NIMS Data Service; Q7: Willingness to try the NIMS Data Service; Q8: Perceived misuse and abuse of prescription medication; Q9: Awareness of dosage increase since initiation.
