# Supplementary material for: Perceptual Discrepancies of Opioid Analgesics and Psychotropic Drugs: A Cross-Sectional Study of Korean Patients and Physicians
Source: J Clin Med. 2025 Oct 31;14(21):7734. doi: 10.3390/jcm14217734 (PMC12609944; doi:10.3390/jcm14217734)
Supplement: Supplementary file 1 [file jcm-14-07734-s001.zip › Supplementary_Materials_S1_Patient_Survey_Questions.pdf]

Perceptual Discrepancies of Opioid Analgesics and Psychotropic Drugs: A Cross-Sectional Study of Korean Patients and Physicians

**Patient Information**			
Study Date		Attending Physician	
Sex/Age		Patient ID	

Explanation and Consent Form for Research Participants

Research Subject Explanation	
Research Project Title	Perceptual Discrepancies of Opioid Analgesics and Psychotropic Drugs: A Cross-Sectional Study of Korean Patients and Physicians
Principal Investigator	Jung Eun Kim

1. Overview

This study is a survey on the current status of the use of opioid analgesics and psychotropic drugs (classified as medical narcotics, previously considered psychotropic substances).

The principal investigator of this study is Jung Eun Kim of Hallym University Kangnam Sacred Heart Hospital (contact: 02-1577-5587). This study will be conducted only for those who voluntarily express their intention to participate. Before deciding to participate, it is important that you understand the purpose of the study, how your information will be used, what is involved in the study, and the potential benefits, risks, and discomforts. Please read the following information carefully and take your time. If you have any questions, please contact the research staff.

2. Background and Purpose of the Study

The basic purpose of the medical narcotics management policy is to establish a system that allows patients who need medical narcotics to use them more safely for therapeutic purposes and to prepare institutional measures to reduce the possibility of illicit use and misuse of medical narcotics. Medical narcotics are not illicit drugs and are essential in clinical settings, but they carry risks of misuse and abuse, raising concerns for public health.

All information related to the prescription of opioid analgesics and psychotropic drugs classified as medical narcotics under the Narcotics Control Act is mandatorily entered into the Narcotics Information Management System (NIMS). The purposes of this study are: 1. To evaluate the perception differences of narcotics between patients and physicians, and 2. To understand patients' and physicians' perceptions of the Narcotics Information Management System (NIMS). Through this, we aim to contribute to effective medical narcotics management and the improvement of national health.

3. Research Participants

We will survey 400 patients who have been taking opioid analgesics or psychotropic drugs (corresponding to Article 2, Paragraph 3, Subparagraph (d) of the Narcotics Control Act) for a long period (more than 180 days within the last year). Patients under 18 years of age, those who have taken opioid analgesics or psychotropic drugs for less than 180 days, patients taking medication for cancer pain control, patients with unstable conditions, or those who refuse to participate will be excluded from the study.

4. Research Methods

Data collection through questionnaires. The estimated time required to complete the questionnaire is approximately 5-10 minutes.

5. Benefits of Research Participation

There will be no financial compensation or direct benefits for your participation in this study. However, all information you provide will help establish future medical narcotics management policies in South Korea.

6. Side Effects or Risks and Discomforts Associated with Research Participation

We do not anticipate any risks or harm to you related to your participation in the study. However, there may be discomforts such as the time required for questionnaire participation and anxiety about the exposure of collected information.

If you have any questions about potential side effects or risks that may occur during your participation in the study, please contact the research staff immediately.

7. Withdrawal from the Study

Your participation in this study is voluntary, and you may withdraw from participation at any time. Furthermore, even if you decide not to participate or withdraw midway, you will not experience any disadvantages. If you wish to withdraw, please inform the research staff immediately.

8. Continuous Provision of New Research-Related Information

I understand that if I have any questions about the details of the study, I can contact the research staff at any time, and that I will be immediately informed if any new information that may affect my participation in this study becomes available.

9. Compensation Measures for Subjects in Case of Harm (Medical Treatment/Compensation)

We will do our best to ensure your safety during the study period.

10. Personal Information and Confidentiality

This study does not collect the names of research subjects who voluntarily participate. If you agree to participate in the study, your general information such as sex and age, and all information collected through the survey tool, will be coded and stored so that only two research staff members can access it. Coded information will be managed through the use of passwords and locking devices. However, for proper research progress verification and oversight, the Institutional Review Board and the Patient Protection Center may review the collected information. Research data will be stored for 3 years after the study ends, and to maintain confidentiality, computer files will be completely deleted and paper documents will be shredded and discarded. By signing this consent form, you grant permission for the above.

11. Voluntary Participation and Withdrawal

Your participation in this study is up to you. You are free not to participate in this study, and there will be no disadvantages to you if you do not participate. Furthermore, even if you decide to participate in the study, you can stop at any time. You will not receive any disadvantages if you do not participate in this study. If you wish to stop participating in the study, please inform the research staff or principal investigator immediately. If you stop participating, your data will no longer be used for the study, and computer files will be completely deleted and paper documents will be shredded and discarded.

12. Responsible Person and Contact Information for Study

If you have any questions about this study or if any problems related to the study arise, please contact the researcher below.

Researcher: Jung Eun Kim

Affiliation: Department of Anesthesiology and Pain Medicine, Hallym University Kangnam Sacred Heart Hospital

Address: Department of Anesthesiology and Pain Medicine, Hallym University Kangnam Sacred Heart Hospital, 1 Singil-ro, Yeongdeungpo-gu, Seoul

Phone Number: Jung Eun Kim **02-1577-5587**

Clinical Research Ethics Committee Phone Number: 02-829-5527

**Consent Form**	
Research Project Title	Perceptual Discrepancies of Opioid Analgesics and Psychotropic Drugs: A Cross-Sectional Study of Korean Patients and Physicians
Principal Investigator	Jung Eun Kim

1. I have received an oral explanation of the study, read the above research subject explanation, and discussed it with the research staff.

2. I have been informed of the risks and benefits and have received satisfactory answers to my questions.

3. I voluntarily agree to participate in this study.

4. I understand that I can refuse to participate in the study or withdraw from participation at any time without affecting future treatment, and that this decision will not harm me.

5. By signing this explanation and consent form, I agree to the collection and processing of my personal information by the researcher for medical research purposes within the scope permitted by current laws and regulations.

6. I understand that I will receive a copy of this consent form.

**Research Subjects**	Name :	Sign :	Date :
**Consent Holder**	Name :	Sign :	Date :

Questions for Patients Only

A. Understanding of the Narcotics Information Management System (NIMS)

1. Do you know the difference between narcotics (Korean: *mayak-ryu*) and drugs (Korean: *mayak*)?
   1) Yes, I know.
   2) No.

2. Are you aware that most opioid analgesics and psychotropic drugs are managed as medical narcotics (formerly psychotropic substances) under the Narcotics Control Act?
   1) Yes, I am aware.
   2) No, I just learned this.

3. Under the Narcotics Control Act, all personal information and prescription information of patients using medical narcotics (formerly psychotropic substances) among opioid analgesics and psychotropic drugs are reported to the Narcotics Information Management System (NIMS) regardless of insurance coverage, and Resident Registration Number is a mandatory collection item for medical narcotics - opioid analgesics, and psychotropic drugs - prescriptions. Are you aware of this fact?
   1) Yes, I am aware.
   2) No, I just learned this.

4. Furthermore, according to Article 18-2 of the Medical Service Act and Article 11-4, Paragraph 2 of the Narcotics Control Act, physicians can use the collected Resident Registration Number to view a patient's past usage history of opioid analgesics and psychotropic drugs. In this case, physicians can view it with notification only (without patient consent). Are you aware of this fact?
   1) Yes, I am aware.
   2) No, I just learned this.

5. If misuse or abuse of medical narcotics is suspected, physicians can legally refuse to prescribe medical narcotics. Are you aware of this fact?

    1) Yes, I am aware.

    2) No, I just learned this.

6. Furthermore, under the same Act, patients can check their own medical narcotics use history through the "My Medication History Inquiry" service (NIMS Data Service; data.nims.or.kr). Are you aware of this?

    1) Yes, I am aware.

    2) No, I just learned this.

7. Are you willing to use the "My Medication History Inquiry" service (NIMS Data Service; data.nims.or.kr)?

    1) Yes, I am willing to use it.

    2) No, I am not willing to use it.

B. Awareness of Your Own Medication

1. Are you aware that you are taking medication designated as medical narcotics under the Narcotics Control Act?
   1) Yes, I am aware.
   2) No, I just learned this.

2. Do you know the name of the medical narcotics you are taking?
   1) Yes, I know. (Please write the name of the drug)

   2) No.

3. How long have you been taking these medical narcotics?
   1) More than 6 months, up to 12 months
   2) More than 12 months, up to 36 months
   3) More than 36 months, up to 60 months
   4) More than 60 months

4. Has the dose (amount, number of types) of opioid analgesics or psychotropic drugs prescribed increased compared to the initial dose?

1) Yes, it has increased.

2) No, I am maintaining the initial dose.

3) I don’t know.

5. Do you think you are misusing or abusing the medications you are currently taking?

1) Yes.

2) No.

Thank you.
