# Supplementary material for: Perceptual Discrepancies of Opioid Analgesics and Psychotropic Drugs: A Cross-Sectional Study of Korean Patients and Physicians
Source: J Clin Med. 2025 Oct 31;14(21):7734. doi: 10.3390/jcm14217734 (PMC12609944; doi:10.3390/jcm14217734)
Supplement: Supplementary file 1 [file jcm-14-07734-s001.zip › Supplementary_Materials_S2_Physician_survey_Questions.pdf]

Perceptual Discrepancies of Opioid Analgesics and Psychotropic Drugs: A Cross-Sectional Study of Korean Patients and Physicians

**Participant Information**			
Name (English Initial)		Sex/Age	

Explanation and Consent Form for Research Participants

**Research Subject Explanation**	
Research Project Title	Perceptual Discrepancies of Opioid Analgesics and Psychotropic Drugs: A Cross-Sectional Study of Korean Patients and Physicians
Principal Investigator	Jung Eun Kim

1. Overview

This study is a survey on the current status of the use of opioid analgesics and psychotropic drugs (classified as medical narcotics, previously considered psychotropic substances).

The principal investigator of this study is Jung Eun Kim of Hallym University Kangnam Sacred Heart Hospital (contact: 02-1577-5587). This study will be conducted only for those who voluntarily express their intention to participate. Before deciding to participate, it is important that you understand the purpose of the study, how your information will be used, what is involved in the study, and the potential benefits, risks, and discomforts. Please read the following information carefully and take your time. If you have any questions, please contact the research staff.

2. Background and Purpose of the Study

The basic purpose of the medical narcotics management policy is to establish a system that allows patients who need medical narcotics to use them more safely for therapeutic purposes and to prepare institutional measures to reduce the possibility of illicit use and misuse of medical narcotics. Medical narcotics are not illicit drugs and are essential in clinical settings, but they carry risks of misuse and abuse, raising concerns for public health.

All information related to the prescription of opioid analgesics and psychotropic drugs classified as medical narcotics under the Narcotics Control Act is mandatorily entered into the Narcotics Information Management System (NIMS). The purposes of this study are: 1. To evaluate the perception differences of narcotics between patients and physicians, and 2. To understand patients' and physicians' perceptions of the Narcotics Information Management System (NIMS). Through this, we aim to contribute to effective medical narcotics management and the improvement of national health.

3. Research Participants

With the cooperation of the Korean Medical Association, we will conduct an email survey targeting 800 physicians from the departments of Anesthesiology and Pain Medicine, Neurology, and Psychiatry with experience prescribing opioid analgesics or psychotropic drugs. Physicians without experience in prescribing medical narcotics will be excluded from the study.

4. Research Methods

Responses are anonymous. The estimated time required to complete the questionnaire is approximately 5-10 minutes.

5. Benefits of Research Participation

There will be no financial compensation or direct benefits for your participation in this study.

However, all information you provide will help establish future medical narcotics management policies in South Korea.

6. Side Effects or Risks and Discomforts Associated with Research Participation

We do not anticipate any risks or harm to you related to your participation in the study. However, there may be discomforts such as the time required for questionnaire participation and anxiety about the exposure of collected information.

If you have any questions about potential side effects or risks that may occur during your participation in the study, please contact the research staff immediately.

7. Withdrawal from the Study

Your participation in this study is voluntary, and you may withdraw from participation at any time. Furthermore, even if you decide not to participate or withdraw midway, you will not experience any disadvantages. If you wish to withdraw, please inform the research staff immediately.

8. Continuous Provision of New Research-Related Information

I understand that if I have any questions about the details of the study, I can contact the research staff at any time, and that I will be immediately informed if any new information that may affect my participation in this study becomes available.

9. Compensation Measures for Subjects in Case of Harm (Medical Treatment/Compensation)

We will do our best to ensure your safety during the study period.

10. Personal Information and Confidentiality

This study does not collect the names of research subjects who voluntarily participate. If you

agree to participate in the study, your general information such as sex and age, and all information collected through the survey tool, will be coded and stored so that only two research staff members can access it. Coded information will be managed through the use of passwords and locking devices. However, for proper research progress verification and oversight, the Institutional Review Board and the Patient Protection Center may review the collected information. Research data will be stored for 3 years after the study ends, and to maintain confidentiality, computer files will be completely deleted and paper documents will be shredded and discarded.

11. Voluntary Participation and Withdrawal

Your participation in this study is up to you. Furthermore, even if you decide to participate in the study, you can stop at any time. If you stop participating, your data will no longer be used for the study, and computer files will be completely deleted.

12. Responsible Person and Contact Information for Study

If you have any questions about this study or if any problems related to the study arise, please contact the researcher below.

Researcher: Jung Eun Kim<br>Affiliation: Department of Anesthesiology and Pain Medicine, Hallym University Kangnam Sacred Heart Hospital<br>Address: Department of Anesthesiology and Pain Medicine, Hallym University Kangnam Sacred Heart Hospital, 1 Singil-ro, Yeongdeungpo-gu, Seoul<br>Phone Number: Jung Eun Kim **02-1577-5587**<br>Clinical Research Ethics Committee Phone Number: 02-829-5527

**Consent Form**	
Research Project Title	Perceptual Discrepancies of Opioid Analgesics and Psychotropic Drugs: A Cross-Sectional Study of Korean Patients and Physicians
Principal Investigator	Jung Eun Kim

1. I have received an explanation of the study, read the above research subject explanation.

2. I have been informed of the risks and benefits and have received satisfactory answers to my questions.

3. I voluntarily agree to participate in this study.

4. I understand that I can refuse to participate in the study or withdraw from participation at any time.

5. By checking this consent form, I agree to allow my responses to be used for research purposes.

   ☐ I agree that the responses in this survey may be used for research purposes.

Questions for Physician Only

A. Participant Information

1. Do you know the difference between narcotics (Korean: *mayak-ryu*) and drugs (Korean: *mayak*)?

    1) Yes, I know.

    2) No.

2. Which of the following statements most closely matches your definition of narcotics misuse or abuse?

    1) Uses beyond the Ministry of Food and Drug Safety (MFDS)-approved label

    2) Excludes uses that are beyond the MFDS-approved label but are widely accepted in textbooks

    3) Excludes uses that are beyond the MFDS-approved label but are prescribed by a physician

    4) Allows uses beyond the MFDS-approved label but prescribed by a physician

3. Do you think the scope of permitted use of medical narcotics should be expanded?

    1) Abuse or misuse of medical narcotics is a serious societal problem, so its use should be restricted to a certain degree.

    2) Although measures to prevent societal misuse and abuse should be established, prescription should be at the discretion of the prescribing physician.

    3) Even if societally perceived as abuse or misuse, use of narcotics should be permitted if there is clear medical necessity.

    4) No response.

4. Do you consider the patient you most recently prescribed medical narcotics to be a patient with medical narcotics misuse or abuse?

    1) Yes.

    2) No.

5. Has the medical narcotics dosage (including number of items) administered to the patient increased in the past year?

    1) Yes.

    2) No.

B. Understanding of Narcotics Information Management System (NIMS)

1. Are you aware that most opioid analgesics and psychotropic drugs prescribed to this patient are managed as medical narcotics (formerly psychotropic substances) under the Narcotics Control Act?

    1) Yes, I am aware.

    2) No, I just learned this.

2. Under the Narcotics Control Act, all personal information and prescription information of patients using medical narcotics (formerly psychotropic substances) among opioid analgesics and psychotropic drugs are reported to the Narcotics Information Management System (NIMS) regardless of insurance coverage, and Resident Registration Number is a mandatory collection item for medical narcotics, opioid analgesics, and psychotropic drugs prescriptions. Are you aware of this fact?

    1) Yes, I am aware.

    2) No, I just learned this.

3. Furthermore, according to Article 18-2 of the Medical Service Act and Article 11-4, Paragraph 2 of the Narcotics Control Act, physicians can use the collected Resident Registration Number to view a patient's past usage history of opioid analgesics and psychotropic drugs. In this case, physicians can view it with notification only (without patient consent). Are you aware of this fact?

    1) Yes, I am aware.

    2) No, I just learned this.

4. If misuse or abuse of medical narcotics is suspected, physicians can legally refuse to prescribe medical narcotics. Are you aware of this fact?

    1) Yes, I am aware.

    2) No, I just learned this.

5. Were you aware of the "My Medication History Inquiry" service (NIMS Data Service; data.nims.or.kr) before participating in this questionnaire?

    1) Yes, I am aware.

    2) No, I just learned this.

6. Are you willing to use the "My Medication History Inquiry" service (NIMS Data Service; data.nims.or.kr)?

    1) Yes, I am willing to use it.

    2) No, I am not willing to use it.

C. Information about You

1. What is your sex and age?

1) Male/Female

2) Age:

2. What is your specialty and year of specialty certification?

1) Anesthesiology and Pain Medicine

2) Neurology

3) Psychiatry

Year of specialty certification (   ).

3. What is your employment type?

1) Private Clinic

2) Employed Physician

3) University Hospital

Thank you.
