# Supplementary material for: Perceptual Discrepancies of Opioid Analgesics and Psychotropic Drugs: A Cross-Sectional Study of Korean Patients and Physicians
Source: J Clin Med. 2025 Oct 31;14(21):7734. doi: 10.3390/jcm14217734 (PMC12609944; doi:10.3390/jcm14217734)
Supplement: Supplementary file 1 [file jcm-14-07734-s001.zip › Supplementary_Table_S1.pdf]

**Supplementary Table S1.** Internal Consistency and Corrected Item-Total Correlations for Questionnaire Subscales by Respondent Group

| Group | Scale (Items) | Item | Corrected Item–Total Correlation | Cronbach's α |
|---|---|---|---|---|
| Patients ($n$ = 322) | Knowledge and Awareness (Q1–Q3) | Q1 | 0.27 | 0.56 |
| | | Q2 | 0.43 | |
| | | Q3 | 0.83 | |
| | System Accessibility (Q4–Q7) | Q4 | 0.32 | 0.61 |
| | | Q5 | 0.37 | |
| | | Q6 | 0.38 | |
| | | Q7 | 0.50 | |
| | Misuse and Abuse (Q8–Q9) | Q8 | -0.02 | -0.04 |
| | | Q9 | -0.02 | |
| Physicians ($n$ = 300) | Knowledge and Awareness (Q1–Q3) | Q1 | -0.03 | -0.02 |
| | | Q2 | 0.02 | |
| | | Q3 | -0.01 | |
| | System Accessibility (Q4–Q7) | Q4 | 0.60 | 0.59 |
| | | Q5 | 0.32 | |
| | | Q6 | 0.60 | |
| | | Q7 | 0.05 | |
| | Misuse and Abuse (Q8–Q9) | Q8 | -0.01 | -0.01 |
| | | Q9 | -0.01 | |

Note: Corrected item–total correlation refers to the correlation between an individual item and the sum of the remaining items in its scale. Cronbach's α values below 0.70, along with low or negative item–total correlations, indicate that items do not form a reliable unidimensional scale and are better analyzed independently as distinct formative indicators.
