# Supplementary material for: Perceptual Discrepancies of Opioid Analgesics and Psychotropic Drugs: A Cross-Sectional Study of Korean Patients and Physicians
Source: J Clin Med. 2025 Oct 31;14(21):7734. doi: 10.3390/jcm14217734 (PMC12609944; doi:10.3390/jcm14217734)
Supplement: Supplementary file 1 [file jcm-14-07734-s001.zip › Supplementary_Table_S2.pdf]

**Supplementary Table S2.** Expanded version of Age-stratified analysis of survey responses (Yes responses only)

| | Patient <60 ($n$ = 228) | Physician <60 ($n$ = 276) | Patient <60 Proportion 95% CI | Physician <60 Proportion 95% CI | Proportion Difference 95% CI | $p$-value (FDR) | Patient ≧60 ($n$ = 92) | Physician ≧60 ($n$ = 24) | Patient ≧60 Proportion 95% CI | Physician ≧60 Proportion 95% CI | Proportion Difference 95% CI | $p$-value (FDR) |
|---|---|---|---|---|---|---|---|---|---|---|---|---|
| Q1 | 23 (10.1) | 160 (58.0) | 10.1 (6.5–14.8) | 58.0 (51.9–63.9) | 47.9 (40.9–54.9) | <0.001 | 9 (9.6) | 16 (66.7) | 9.6 (4.5–17.4) | 66.7 (44.7–84.4) | 57.1 (37.3–76.9) | <0.001 |
| Q2 | 127 (55.7) | 249 (90.2) | 55.7 (49.0–62.3) | 90.2 (86.1–93.5) | 34.5 (27.2–41.9) | <0.001 | 40 (42.6) | 23 (95.8) | 42.6 (32.4–53.2) | 95.8 (78.9–99.9) | 53.3 (40.5–66.1) | <0.001 |
| Q3 | 82 (36.0) | 219 (79.3) | 36.0 (29.7–42.6) | 79.3 (74.1–84.0) | 34.5 (27.2–41.9) | <0.001 | 28 (29.8) | 21 (87.5) | 29.8 (20.8–40.1) | 87.5 (67.6–97.3) | 57.7 (41.6–73.9) | <0.001 |
| Q4 | 68 (29.8) | 100 (36.2) | 29.8 (24.0–36.2) | 36.2 (30.6–42.2) | 6.4 (-1.8–14.6) | 0.0.166 | 19 (20.2) | 12 (50.0) | 20.2 (12.6–29.8) | 50.0 (29.1–70.9) | 29.8 (8.2–51.4) | 0.004 |
| Q5 | 117 (51.3) | 220 (79.7) | 51.3 (44.6–58.0) | 79.7 (74.5–84.3) | 28.4 (20.4–36.4) | <0.001 | 51 (54.3) | 23 (95.8) | 54.3 (43.7–64.6) | 95.8 (78.9–99.9) | 41.6 (28.7–54.4) | <0.001 |
| Q6 | 36 (15.8) | 90 (32.6) | 15.8 (11.3–21.2) | 32.6 (27.1–38.5) | 16.8 (9.5–24.1) | <0.001 | 11 (11.7) | 13 (54.2) | 11.7 (6.0–20.0) | 54.2 (32.8–74.4) | 42.5 (21.5–63.4) | <0.001 |
| Q7 | 102 (44.7) | 107 (38.8) | 44.7 (38.2–51.4) | 38.8 (33.0–44.8) | 6.0 (-2.7–14.6) | 0.198 | 33 (35.1) | 13 (54.2) | 35.1 (25.5–45.6) | 54.2 (32.8–74.4) | 19.1 (-3.1–41.2) | 0.098 |
| Q8 | 183 (80.3) | 29 (10.5) | 80.3 (74.5–85.2) | 10.5 (7.2–14.7) | 69.8 (63.4–76.1) | <0.001 | 70 (74.5) | 1 (4.2) | 74.5 (64.4–82.9) | 4.2 (0.1–21.1) | 70.3 (58.4–82.2) | <0.001 |
| Q9 | 38 (16.7) | 48 (17.4) | 16.7 (12.1–22.2) | 17.4 (13.1–22.4) | 0.7 (-5.9–7.3) | 0.830 | 14 (14.9) | 4 (16.7) | 14.9 (8.4–23.7) | 16.7 (4.7–37.4) | 1.8 (-14.8–18.3) | 0.760 |

Values are counts and percentages of 'Yes' responses; No responses omitted. Percentages calculated using group-specific denominators. Chi-square tests used for comparisons with each age stratum. The ≥60 physician stratum ($n$ = 24) showed wider CIs, warranting cautious interpretation due to the smaller sample size.

Q1: Distinguishing medical narcotics from illicit drugs; Q2: Awareness that prescribed medications are classified as medical narcotics; Q3: Awareness of the NIMS reporting; Q4: Awareness of the narcotics prescription status inquiry system; Q5: Awareness of physician's right to refuse prescription; Q6: Awareness of the NIMS Data Service; Q7: Willingness to try the NIMS Data Service; Q8: Perceived misuse and abuse of prescription medication; Q9: Awareness of dosage increase since initiation.
