# Supplementary material for: Perceptual Discrepancies of Opioid Analgesics and Psychotropic Drugs: A Cross-Sectional Study of Korean Patients and Physicians
Source: J Clin Med. 2025 Oct 31;14(21):7734. doi: 10.3390/jcm14217734 (PMC12609944; doi:10.3390/jcm14217734)
Supplement: Supplementary file 1 [file jcm-14-07734-s001.zip › Supplementary_Table_S3.pdf]

Supplementary Table S3. Expanded version of Correlation analysis with Patient's Education (Yes responses only)

Survey Questions		Education				Overall *p*-value	Post hoc test (Bonferroni's correction)					
		HL	AD	BD	GD		HL vs. AD	HL vs. BD	HL vs. GD	AD vs. BD	AD vs. GD	BD vs. GD
Narcotics Knowledge and Awareness, *n* (%)	Q1: 32 (9.9)	0 (0.0)	1 (1.4)	25 (13.8)	6 (46.2)	<0.001	>0.999	0.017	<0.001	0.023	<0.001	0.047
	Q2: 167 (51.9)	7 (12.1)	22 (31.4)	126 (69.6)	12 (92.3)	<0.001	0.055	<0.001	<0.001	<0.001	<0.001	0.682
	Q3: 110 (34.2)	6 (10.3)	15 (21.4)	79 (43.6)	10 (76.9)	<0.001	0.551	<0.001	<0.001	0.007	0.001	0.120
Narcotics Control System Accessibility, *n* (%)	Q4: 87 (27.0)	5 (8.6)	13 (18.6)	64 (35.4)	5 (38.5)	<0.001	0.642	<0.001	0.087	0.058	0.858	>0.999
	Q5: 168 (52.2)	28 (48.3)	28 (40.0)	105 (58.0)	7 (53.8)	0.072	>0.999	>0.999	>0.999	0..062	>0.999	>0.999
	Q6: 47 (14.6)	2 (3.4)	4 (5.7)	36 (19.9)	5 (38.5)	<0.001	>0.999	0.017	0.010	0.036	0.023	0.918
	Q7: 135 (41.9)	18 (31.0)	21 (30.0)	91 (50.3)	5 (38.5)	0.007	>0.999	0.063	>0.999	0.023	>0.999	>0.999
Misuse and Abuse, *n* (%)	Q8: 253 (78.6)	45 (77.6)	60 (85.7)	138 (76.2)	10 (76.9)	0.430	>0.999	>0.999	>0.999	0.595	>0.999	>0.999
	Q9: 52 (16.1)	11 (19.0)	6 (8.6)	34 (18.8)	1 (7.7)	0.178	0.507	>0.999	>0.999	0.285	>0.999	>0.999

Values are counts and percentages of "Yes" responses; No responses omitted. Total patients *n* = 322. Data presented as *n* (%). NIMS: Narcotics Information Management System. Chi-square tests (Fisher's exact test when appropriate) used for comparisons.
Q1: Distinguishing medical narcotics from illicit drugs; Q2: Awareness that prescribed medication is medical narcotics; Q3: Awareness of the NIMS reporting; Q4: Awareness of the narcotics prescription status inquiry system; Q5: Awareness of physician's right to refuse prescription; Q6: Awareness of the NIMS Data Service; Q7: Willing to try the NIMS Data Service; Q8: Perceptions of prescription medication use; Q9: Awareness of dosage increase from initial, HL: high school or less; AD: Associate degree; BD: Bechalor's degree; GD: Graduate degree
