# Supplementary material for: Perceptual Discrepancies of Opioid Analgesics and Psychotropic Drugs: A Cross-Sectional Study of Korean Patients and Physicians
Source: J Clin Med. 2025 Oct 31;14(21):7734. doi: 10.3390/jcm14217734 (PMC12609944; doi:10.3390/jcm14217734)
Supplement: Supplementary file 1 [file jcm-14-07734-s001.zip › Supplementaty_Table_S4.pdf]

Supplementary Table S4. Detailed subgroup analysis by patient treatment duration: Multivariable logistic regression

Category	Subgroup	Outcome	OR (95% CI)	*p*-value	Predicted Probability (95% CI)		Risk Difference (95% CI)	Hosmer-Lemeshow Test (goodness-of-fit)
					Patient	Physician		
Patient Subgroup	Patient: Treatment duration 6–12 months (*n* = 90)	Q1	20.41 (7.74–53.84)	<0.001	4.8 (-4.8–14.5)	60.7 (54.3–67.1)	55.9 (43.2–68.5)	0.793
		Q2	6.15 (2.83–13.36)	<0.001	61.9 (51.3–72.4)	90.7 (86.5–94.9)	28.8 (16.8–40.7)	0.379
		Q3	6.29 (3.19–12.41)	<0.001	41.4 (29.7–53.1)	80.2 (74.5–85.9)	38.8 (24.8–52.7)	0.305
		Q4	1.00 (0.53–1.88)	0.995	36.6 (25.1–48.0)	36.2 (29.2–43.2)	0.3 (-14.4–15.0)	0.985
		Q5	3.57 (1.83–6.94)	<0.001	53.0 (41.5–64.5)	79.4 (73.7–85.2)	26.5 (12.6–40.4)	0.974
		Q6	2.70 (1.25–5.83)	0.018	16.2 (5.7–26.8)	35.8 (29.1–42.4)	19.5 (5.6–33.4)	0.807
		Q7	1.13 (0.61–2.10)	0.775	40.0 (28.0–52.0)	43.5 (36.3–50.7)	3.5 (-11.8–18.8)	0.186
		Q8	0.04 (0.02–0.09)	<0.001	74.9 (64.7–85.1)	12.0 (6.9–17.2)	62.9 (50.6–75.1)	0.251
		Q9	1.22 (0.55–2.70)	0.775	17.3 (8.9–25.7)	19.8 (14.2–25.4)	2.5 (-7.9–12.9)	0.889
	Patient: Treatment duration >12–36 months (*n* = 123)	Q1	22.58 (10.06–50.65)	<0.001	6.2 (2.0–10.5)	59.4 (53.2–65.5)	53.1 (45.5–60.8)	0.76
		Q2	9.33 (5.18–16.81)	<0.001	49.5 (40.7–58.4)	90.9 (86.5–95.3)	41.4 (31.2–51.5)	0.037
		Q3	9.02 (5.27–15.47)	<0.001	30.9 (22.6–39.2)	79.8 (74.3–85.3)	48.9 (38.5–59.3)	0.647
		Q4	1.36 (0.82–2.27)	0.259	29.6 (21.4–37.7)	36.4 (29.8–43.0)	6.9 (-4.3–18.0)	0.605
		Q5	3.10 (1.86–5.18)	<0.001	55.1 (46.3–64.0)	78.9 (73.0–84.7)	23.7 (12.7–34.8)	0.387
		Q6	3.76 (1.97–7.17)	<0.001	13.0 (7.1–19.0)	33.9 (27.7–40.1)	20.9 (11.9–29.9)	0.643
		Q7	1.00 (0.61–1.61)	0.985	39.6 (30.8–48.5)	40.1 (33.5–46.7)	0.5 (-10.9–11.8)	0.239
		Q8	0.03 (0.01–0.05)	<0.001	81.6 (74.7–88.5)	11.2 (6.8–15.7)	70.4 (61.7–79.1)	0.446
		Q9	1.60 (0.84–3.02)	0.195	14.0 (8.2–19.9)	23.2 (17.6–28.7)	9.1 (1.4–16.9)	0.845
	Patient: Treatment duration >36–60 months (*n* = 76)	Q1	6.63 (3.06–14.35)	<0.001	18.7 (9.2–28.3)	57.8 (50.9–64.8)	39.1 (26.6–51.5)	0.238
		Q2	8.36 (3.94–17.75)	<0.001	48.5 (36.6–60.4)	91.1 (86.5–95.8)	42.6 (29.3–55.9)	0.328
		Q3	6.85 (3.43–13.66)	<0.001	32.3 (20.9–43.7)	76.9 (70.5–83.4)	44.6 (30.4–58.7)	0.894
		Q4	2.03 (0.98–4.19)	0.071	23.0 (12.5–33.5)	36.1 (29.2–43.0)	13.1 (-0.6–26.7)	0.323
		Q5	4.60 (2.32–9.12)	<0.001	47.4 (35.0–59.9)	80.6 (74.8–86.4)	33.2 (18.7–47.7)	0.634
		Q6	2.39 (1.10–5.19)	0.041	19.0 (9.8–28.2)	32.5 (25.9–39.0)	13.5 (1.4–25.5)	0.299
		Q7	0.69 (0.37–1.31)	0.295	48.6 (35.9–61.4)	39.9 (32.9–47.0)	8.7 (-6.8–24.2)	0.7
		Q8	0.03 (0.01–0.08)	<0.001	79.7 (69.3–90.0)	12.6 (7.3–17.9)	67.1 (54.7–79.5)	0.233
		Q9	1.07 (0.47–2.42)	0.875	19.2 (9.6–28.8)	20.7 (14.8–26.6)	1.5 (-10.3–13.2)	0.695
	Patient: Treatment duration >60 months (*n* = 33)	Q1	9.34 (2.94–29.66)	<0.001	13.3 (-2.8–29.4)	60.6 (53.6–67.7)	47.3 (28.6–66.1)	0.93
		Q2	21.42 (5.99–76.63)	<0.001	37.6 (19.7–55.4)	91.8 (87.8–95.7)	54.2 (35.6–72.8)	0.397
		Q3	6.37 (2.20–18.42)	0.001	33.9 (15.0–52.7)	77.9 (71.4–84.3)	44.0 (23.0–65.0)	0.857
		Q4	3.82 (1.15–12.65)	0.037	15.8 (2.3–29.3)	35.1 (28.2–42.1)	19.3 (2.7–36.0)	0.96
		Q5	3.54 (1.22–10.29)	0.030	53.0 (33.4–72.6)	79.6 (73.4–85.8)	26.6 (5.2–48.1)	0.39
		Q6	4.09 (1.27–13.19)	0.030	12.3 (-1.3–26.0)	34.1 (27.4–40.8)	21.8 (5.5–38.2)	0.848
		Q7	0.78 (0.29–2.07)	0.619	45.3 (24.9–65.7)	40.1 (32.9–47.4)	5.2 (-17.5–28.0)	0.501

Q8	0.06 (0.02–0.21)	<0.001	73.3 (57.0–89.6)	12.2 (6.9–17.4)	61.2 (43.1–79.2)	0.706
Q9	7.91 (0.88–71.42)	0.074	7.2 (-14.6–29.0)	22.2 (15.4–29.0)	15.1 (-9.8–40.0)	0.634

Multivariable logistic regression models were adjusted for age and gender. *P*-values were corrected using the false discovery rate (FDR) method to account for multiple comparisons. Patient subgroup analysis included all physician participants (*n* = 300). Treatment duration categories: 6–12 months (*n* = 90), >12–36 months (*n* = 123), >36–60 months (*n* = 76), >60 months (*n* = 33). OR, odds ratio; CI, confidence interval.

Q1: Distinguishing medical narcotics from illicit drugs; Q2: Awareness that prescribed medications are classified as medical narcotics; Q3: Awareness of the NIMS reporting; Q4: Awareness of the narcotics prescription status inquiry system; Q5: Awareness of physician's right to refuse prescription; Q6: Awareness of the NIMS Data Service; Q7: Willingness to try the NIMS Data Service; Q8: Perceived misuse and abuse of prescription medication; Q9: Awareness of dosage increase since initiation.
